# Downregulated Wnt/β-catenin signalling in the Down syndrome hippocampus

**DOI:** 10.1038/s41598-019-43820-4

**Published:** 2019-05-13

**Authors:** Simone Granno, Jonathon Nixon-Abell, Daniel C. Berwick, Justin Tosh, George Heaton, Sultan Almudimeegh, Zenisha Nagda, Jean-Christophe Rain, Manuela Zanda, Vincent Plagnol, Victor L. J. Tybulewicz, Karen Cleverley, Frances K. Wiseman, Elizabeth M. C. Fisher, Kirsten Harvey

**Affiliations:** 1 Department of Pharmacology, UCL School of Pharmacy, University College London, 29-39 Brunswick Square, London, WC1N 1AX UK; 2 Department of Neuromuscular Diseases, UCL Institute of Neurology, Queen Square, London, WC1N 3BG UK; 3 Cell Biology Section, Neurogenetics Branch, National Institute of Neurological Disorders and Stroke (NINDS), Bethesda, MD USA; 4 School of Health, Life and Chemical Sciences, The Open University, Walton Hall, Milton Keynes, MK6 7AA UK; 5 Hybrigenics Services - Fondation Jérôme Lejeune, 3-5 Impasse Reille, 75014 Paris, France; 6 UCL Genetics Institute, Darwin Building, Gower Street, London, WC1E 6BT UK; 7 The Francis Crick Institute, 1 Midland Rd, Kings Cross, London, NW1 1AT UK; 8 Department of Medicine, Imperial College, London, W12 0NN UK; 9 London Down Syndrome Consortium (LonDownS), London, UK

**Keywords:** Developmental disorders, Alzheimer's disease

## Abstract

Pathological mechanisms underlying Down syndrome (DS)/Trisomy 21, including dysregulation of essential signalling processes remain poorly understood. Combining bioinformatics with RNA and protein analysis, we identified downregulation of the Wnt/β-catenin pathway in the hippocampus of adult DS individuals with Alzheimer’s disease and the ‘Tc1’ DS mouse model. Providing a potential underlying molecular pathway, we demonstrate that the chromosome 21 kinase DYRK1A regulates Wnt signalling via a novel bimodal mechanism. Under basal conditions, DYRK1A is a negative regulator of Wnt/β-catenin. Following pathway activation, however, DYRK1A exerts the opposite effect, increasing signalling activity. In summary, we identified downregulation of hippocampal Wnt/β-catenin signalling in DS, possibly mediated by a dose dependent effect of the chromosome 21-encoded kinase DYRK1A. Overall, we propose that dosage imbalance of the Hsa21 gene *DYRK1A* affects downstream Wnt target genes. Therefore, modulation of Wnt signalling may open unexplored avenues for DS and Alzheimer’s disease treatment.

## Introduction

Down syndrome (DS) is the most common human aneuploidy, occurring in approximately 1/700–1000 live births^1^. It is caused by trisomy of chromosome 21 (Hsa21) and is associated with several distinctive characteristics, including intellectual disability and neurodegeneration^2–6^. DS phenotypes likely arise from gene dosage effects resulting from the additional chromosome^7,8^. However, the key cellular mechanisms underlying pathology are poorly understood. Identification of target molecular pathways contributing to the disease is urgently needed to develop effective therapeutic strategies. In this study, we investigated dysfunction of canonical Wnt/β-catenin signalling in DS. The Wnt signalling pathway is a highly conserved signal transduction cascade with high activity during development but of key importance in adulthood as well^9^. Activation of the pathway is dependent upon nuclear translocation of β-catenin, which drives expression of several target genes. Canonical Wnt signalling plays fundamental, well-described roles in several biological processes, including development^10,11^, adult nervous system function^12^, stem cell function^13^ and tumorigenesis^14^.

DS has long been associated with a high incidence of early-onset Alzheimer’s disease (AD-DS)^15–17^. On the other hand, recent evidence has consistently implicated defects in canonical Wnt signalling in the pathogenesis of AD^18–24^. Here, we identified substantial Wnt signalling downregulation in the hippocampus of DS individuals with AD pathology and the Tc1 mouse model of DS. This suggests the presence of a novel functional relationship between DS and Wnt signalling, which may contribute to the development of AD in the ageing DS population. We further characterised such relationship via a candidate gene approach. Wnt dysregulation in DS, in fact, might arise from increased expression of key Hsa21-encoded proteins such as DYRK1A (dual-specificity tyrosine phosphorylation-regulated kinase 1A). In support of this hypothesis, we present evidence of novel bimodal regulatory effects of DYRK1A overexpression, kinase inhibition and interaction with other Wnt signalling components. The kinase DYRK1A has a multitude of substrates regulating developmental, neuronal and neurodegenerative cell signalling mechanisms, and functionally interacts with the primary Wnt components GSK3β and p120/δ-catenin^25–36^. DYRK1A is thought to be an essential mediator of intellectual disability in DS, and has also been proposed to contribute to DS/AD pathogenesis as well as idiopathic AD^37–45^.

## Results

### Wnt signalling is downregulated in human AD-DS

**Figure 1:**
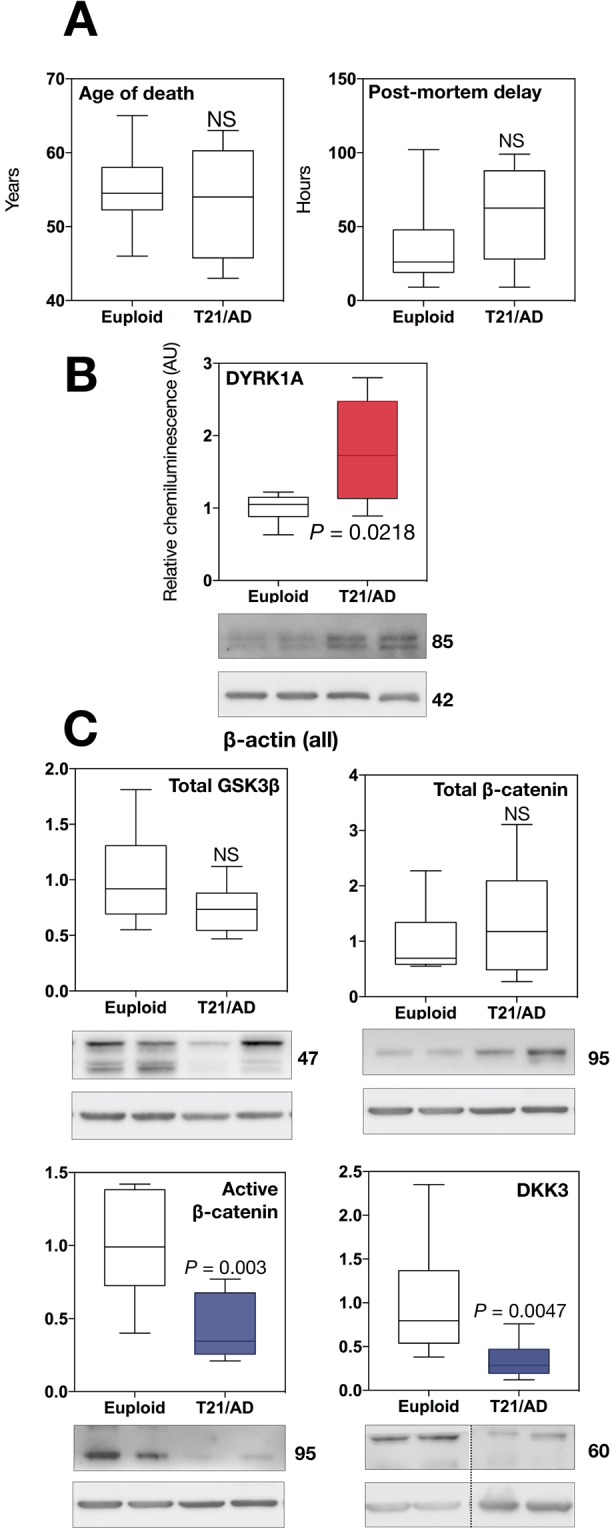
Wnt signalling is altered in human DS. (**A**) Overview of human DS brain samples employed in immunoblot analysis, indicating no significant difference in age of death and post-mortem delay of sample collection between DS and euploid controls. (**B**,**C**) Immunoblot analysis of the human DS hippocampus (n = 8) demonstrating significantly elevated DYRK1A levels (red), as expected, with substantially reduced active β-catenin fraction (blue). DKK3 levels were also significantly reduced (blue).

We first sought to determine whether canonical Wnt signalling alterations are present in the human DS hippocampus. Employing immunoblotting, we characterised activity of the Wnt pathway in post mortem human hippocampal samples from DS adults (Newcastle Brain Bank, average age at death: 56 years; Fig. 1A). Available clinical information indicated the presence of dementia in all patients, with a majority displaying AD neuropathology, typical of DS at this age of death, as assessed by Braak staging (Table S1).

The Hsa21-encoded protein DYRK1A was selected as a marker of Hsa21 trisomy, given its ubiquitous upregulation and key importance in DS^27^. As expected, DYRK1A protein levels were elevated in human hippocampal samples (Fig. 1B, red). In order to biochemically quantify Wnt signalling activity levels, we measured protein amounts of ‘free’ β-catenin, represented by its dephosphorylated form at Ser37/Thr41 and expressed as a fraction of total β-catenin. This is a well established approach^46^. Strikingly, we observed a substantial decrease in hippocampal active β-catenin (~3-fold, *P* = 0.003; Fig. 1C, left blue), whilst levels of total β-catenin remained unaffected. We also detected a significant decrease in hippocampal protein levels of the Wnt inhibitor DKK3 (Fig. 1C, right blue). Total amounts of the key β-catenin inhibitor GSKβ showed no significant differences. Post-mortem human data thus demonstrated suppressed Wnt signalling activity in the DS hippocampus, a novel finding implicating this pathway in DS pathology.

### Wnt signalling is downregulated in the Tc1 mouse hippocampus

**Figure 2:**
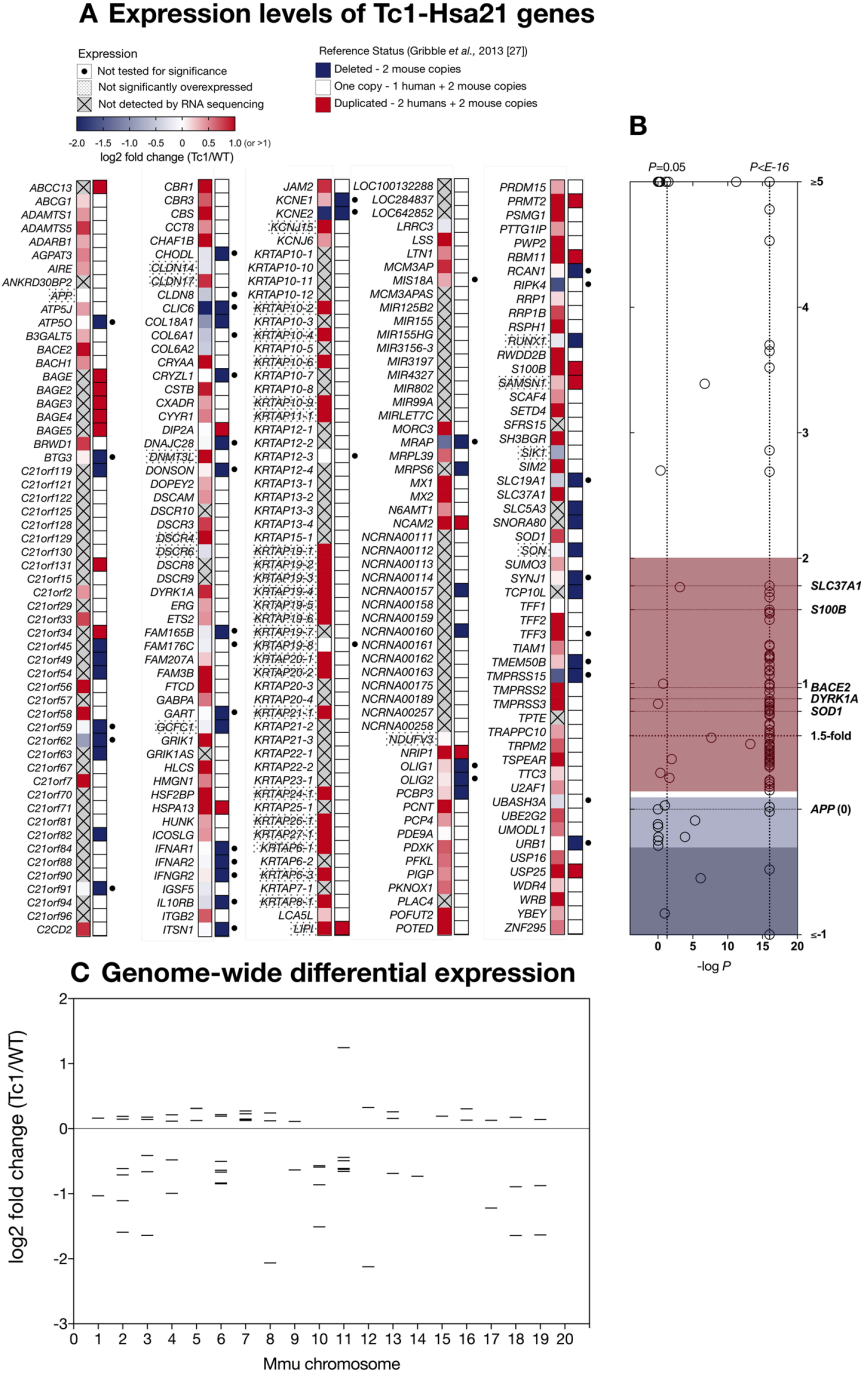
Overview of hippocampal RNAseq in the Tc1 mouse model of DS. (**A**) Hsa21 genes in the Tc1 mouse hippocampus (*n* = 3, adjusted *P* < 0.05), as detected by RNAseq. Overall expression levels of 180 Hsa21 genes which could reliably measured by RNAseq are visually summarised. Of these, 143 could be tested for significance (others highlighted by black circle). Genes are presented alphabetically and colour-coded according to log_2_ fold change/WT. Gene set is matched against a reference list 224 of Hsa21-Tc1 genes published by Gribble *et al*.^48^, providing an overview of expected deletions/duplications (undetected genes present in reference list are crossed out). Copy number refers to Hsa21 genes, with ‘one copy’ indicating modelled triplication of the relevant gene (1 human + 2 endogenous mouse copies). (**B**) Expression levels plotted as a function of significance, highlighting DS/AD-relevant genes, and demonstrating the presence of several genes not functionally overexpressed (blue), including *APP*. (**C**) Expression levels of 64 Tc1 DEX genes (*n* = 3, adjusted *P* < 0.05) beyond Hsa21, arranged by chromosomal number, indicating occurrence of genome-wide, bidirectional transcriptional alterations.

**Figure 3:**
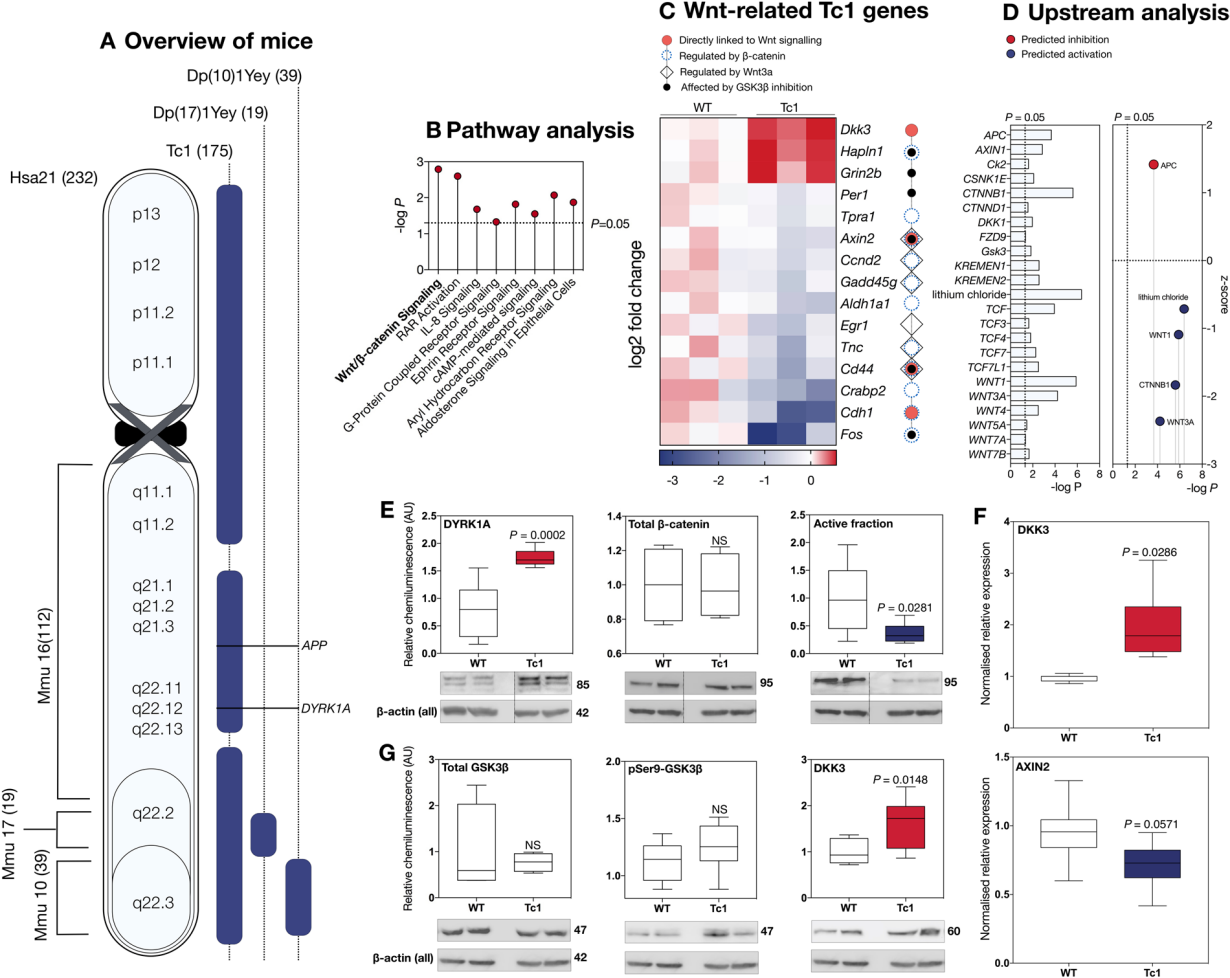
Genetic and biochemical analysis of the Tc1 hippocampus reveals abnormalities in canonical Wnt signalling. (**A**) Overview of DS mouse models employed derived from^50^, highlighting Hsa21 syntenic regions on mouse chromosomes 10, 16 and 17 (left, syntenic gene number in brackets) and trisomic regions in the Tc1^47,48^, Dp(17)1Yey and Dp(17)1Yey (54) strains (right, duplicated gene number in brackets). (**B**) Pathway analysis of the Tc1 dataset. (**C**) Curated list of differentially expressed (DEX), Wnt-related genes for individual Tc1 mice as measured via RNAseq (RNAseq) (*n* = 3; 3 months; adjusted *P* < 0.05). Genes included were identified by Qiagen IPA as either primary Wnt signalling components (red circles) or as regulated by β-catenin (blue dotted circles). Values are expressed as log_2_fold changes. (**D**) IPA Upstream analysis of the Tc1 dataset, revealing several canonical Wnt components as regulators of DEX genes (left panel). Predictive activation z-scores calculated by IPA for upstream regulators with sufficient overlap (right panel) plotting scores as a function of significance (−log_10_*P*). (**E**) Immunoblot analysis of male Tc1 mouse hippocampi (*n* = 8, 160 ± 28 days) compared to age-matched littermates demonstrated enhanced levels of DYRK1A, as expected (red), with reduced canonical Wnt signalling activity (blue). Signal represents the ratio of Ser37/Thr41 dephosphorylated β-catenin to total amounts of β-catenin. (**F**) Quantitative real-time PCR (qPCR) of DKK3 and AXIN2, two DEX Wnt components in the Tc1 hippocampus, demonstrating significantly elevated DKK3 expression (red), and a decreased AXIN2 levels (blue). (**G**) Immunoblot analysis of Tc1 hippocampal samples (*n* = 8) revealed unaltered levels of total and p-Ser9 GSK3β as previously described^52,53^ and enhanced DKK3 protein levels (red).

Having newly identified Wnt signalling downregulation in the human DS hippocampus, we expanded our analysis to the Tc1 mouse, an established DS model^47^, in order to validate this finding. To this end, we performed a combined analysis at the transcriptomic and protein levels. We first employed RNA sequencing (RNAseq) to investigate differential gene expression in the Tc1 hippocampus (Figs 2 and 3; *n* = 3, males, 3 months)^47^. The Tc1 mouse carries a freely-segregating, near-complete Hsa21 (70%, Fig. 3A). In accordance with previous reports, RNAseq analysis indicated that, in this model, not all triplicated genes are dosage-sensitive (Fig. 2A,B) and are thus not equally likely to contribute to phenotypes^47–49^. Additionally, genome-wide transcriptional alterations were observed beyond Hsa21, with 64 differentially expressed (DEX) Tc1 genes (Fig. 2C and Table S2, P < 0.05). This known phenomenon^50^ circumstantially suggests that Hsa21 trisomy might affect molecular pathways regulating global gene expression, such as canonical Wnt signalling.

Given our findings in human hippocampi, as well as the known roles of Wnt signalling in several biological mechanisms likely to be important in DS, such as brain development and AD, we employed QIAGEN Ingenuity® Pathway Analysis (IPA)^51^ to search the DEX gene dataset for Wnt signalling abnormalities. Hsa21 genes were excluded from the analysis, in order to isolate secondary transcriptional effects mediated by trisomy, but not directly associated with overexpression of triplicated genes. In the Tc1 hippocampus, pathway analysis (Fig. 3B) revealed a significant association with canonical Wnt signalling (*P* = 0.00173), with four corresponding DEX genes directly linked to the pathway (Fig. 3C, red circles; *Axin2*, *Cdd4*, *Cdh1*, *Dkk3*). We then performed upstream analysis, probing the IPA knowledge base for regulators potentially responsible for observed expression patterns. This approach detected a further 15 Tc1 DEX genes as significantly regulated by Wnt proteins (Fig. 3C). Interestingly, virtually all Wnt signalling components were identified as significant upstream regulators (Fig. 3D, left panel; Table S3). This finding suggests a high degree of overlap between the Tc1 hippocampal transcriptome and Wnt signalling function, given that IPA linked all essential Wnt signalling components to one or more DEX genes. The transcriptional activator β-catenin (Fig. 3C, dotted circles) and the ligand Wnt3a (squares) were most significantly associated with hippocampal Tc1 expression patterns, regulating expression of 11 and 6 DEX genes, respectively (*P* < 0.0001). Additionally, IPA-generated activation z-scores were overall predictive of significant downregulation of Wnt activity, with negative values registered for several regulators, including β-catenin and Wnt3a (Fig. 3D, right panel). Importantly, these results are consistent with our biochemical data in humans.

Next, we employed immunoblotting to investigate levels of Wnt signalling activity in the Tc1 mouse hippocampus (*n* = 8, 6 months of age; Fig. 3E). The Hsa21-encoded protein DYRK1A was once again selected as a marker of Hsa21 trisomy, based on RNAseq results for duplicated genes (Fig. 2A,B) and its importance in DS^27^. As expected, we found DYRK1A protein levels to be elevated in the Tc1 hippocampus (Fig. 3E, red). In line with human DS hippocampal data, the active β-catenin fraction was significantly reduced ~3-fold (Fig. 3E, blue), whilst total β-catenin remained unaltered. These results were also consistent with IPA predictions, overall suggesting substantial Wnt signalling downregulation in the adult Tc1 mouse hippocampus.

We then assessed Tc1 hippocampal expression of the Wnt inhibitor *Dkk3* and target gene *Axin2*, shown by RNAseq to be up- and downregulated in the Tc1 mouse, respectively. By quantitative PCR (qPCR, Fig. 3F), we found levels consistent with RNAseq data: *Dkk3* expression was significantly elevated, while *Axin2* trended toward decrease (*P* = 0.0571). Consistently, DKK3 protein levels were elevated in the Tc1 hippocampus (Fig. 3G, red). This finding, however, was directionally opposite to what we observed in the human DS hippocampus, where DKK3 protein levels were decreased. We then quantified GSK3β, finding no differences in protein levels (Fig. 3G). The serine 9 (Ser9)-phosphorylated form of GSK3β, expressed as a fraction of total GSK3β was also unaltered, as previously shown in young Tc1 mouse brains^52,53^. Phosphorylation of this residue is however reportedly elevated in aged (20 months) Tc1 hippocampal and cerebral cortical tissue, suggesting a potential age-related effect.

We also probed for total and active β-catenin in the hippocampus of two additional DS models the Dp(10)1Yey and Dp(17)1Yey^54^. These are segmentally trisomic for Mmu10/17 regions of synteny with Hsa21 (Fig. 3A). In these tissues, we found no significant alterations in Wnt activity (Fig. S2). Overall, RNAseq analysis and immunoblotting results were consistent, suggesting significant downregulation of Wnt signalling activity in the Tc1 mouse hippocampus. Since we saw no effects on Wnt signalling in mice carrying triplications of Mmu10 or 17, this novel Tc1 phenotype likely arises from gene(s) with homology to Mmu16.

Our data overall suggest novel Wnt/β-catenin signalling dysfunction in DS, with consistent downregulation observed in both human and Tc1 hippocampi. At this stage, however, we cannot exclude that directional changes may be variable, and tissue-specific.

### DYRK1A interacts with Wnt signalling components

Given these results, we hypothesised that one or more of the Hsa21 genes that are differentially expressed in DS mediated the detected alterations in canonical Wnt signalling. The absence of Wnt signalling changes in the Dp(10)1Yey and Dp(17)1Yey mouse models suggests that Mmu10/17 regions of synteny with Hsa21 are unlikely to influence the phenotypes observed in the Tc1 hippocampus. Thus, candidate Wnt modulators likely map to Mmu16. A number of Hsa21 genes may affect Wnt signalling^31,55–57^, including amyloid precursor protein (*APP*) and *DYRK1A*, which might be particularly important given their roles in DS and AD^27,50^. However, because of the lack of functional *APP* overexpression in the Tc1 mouse^48^ (Fig. 2A,B), this gene is unlikely to primarily mediate hippocampal Wnt phenotypes in this model. Therefore, we selected *DYRK1A* for further study, though we do not exclude that *APP* and/or other Hsa21 genes may also affect Wnt signalling.

**Figure 4:**
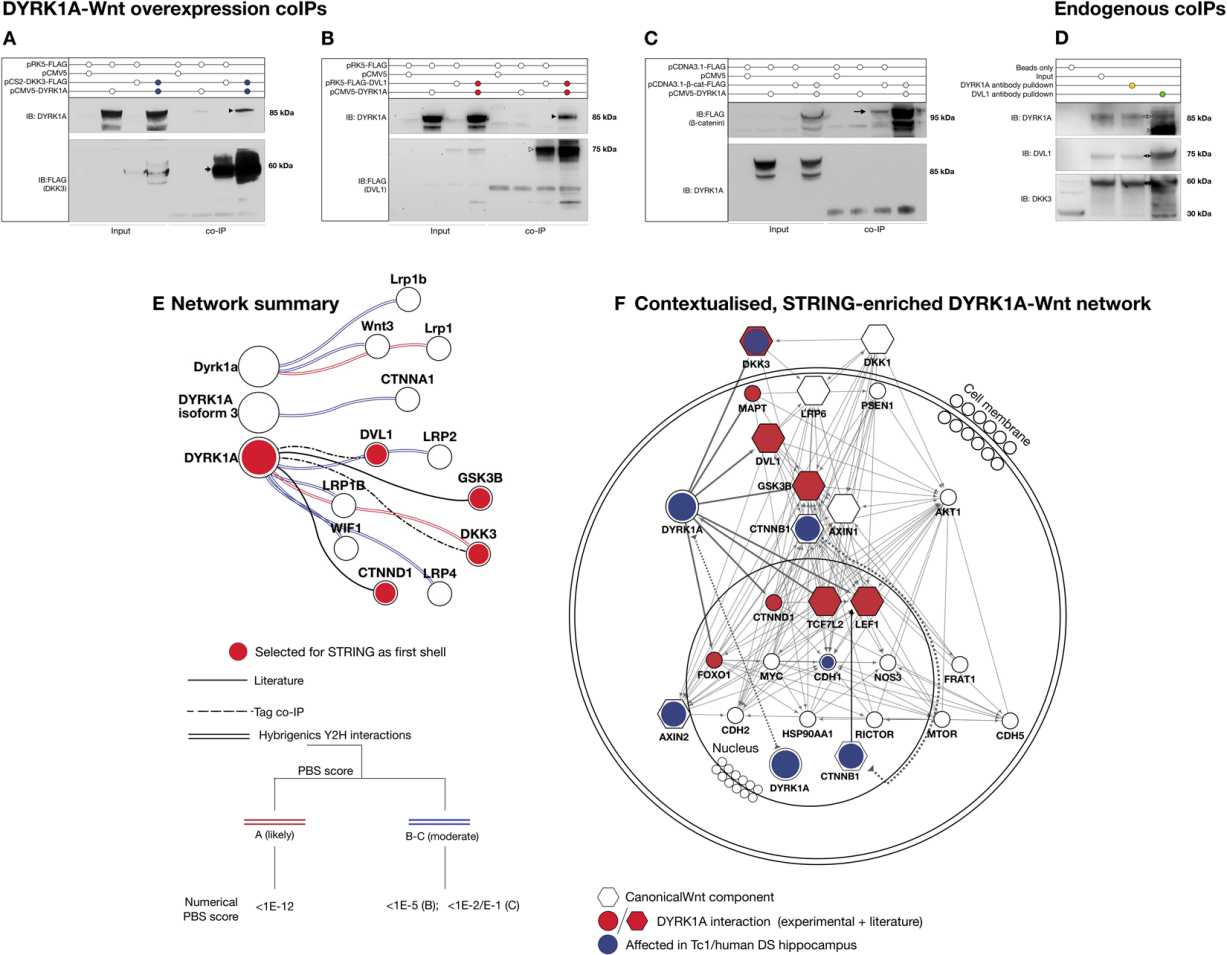
DYRK1A may participate in a highly-interconnected Wnt signalling protein interaction network. (**A**–**C**) Tag co-immunoprecipitation experiments verified via immunoblotting demonstrate a positive interaction between DYRK1A-DKK3 (**A**, blue) and DYRK1A-DVL1 (**B**, red). No direct interaction with β-catenin was observed, as previously described^31^. (**C**) For all co-IPs, 0.5 μg/ml of each construct was transfected for 24 hrs, in three independent repeats. (**D**) Co-immunoprecipitation of DYRK1A-DVL1 and DYRK1A-DKK3 protein complexes in HEK293 cells under endogenous conditions. Complexes were pulled down with magnetic beads coupled to either a DYRK1A (lane 3) or DVL1 antibody (lane 4). Lanes 1 (mock coIP, beads only), 3 and 4 represent results reactions identical conditions save for the antibody employed for pull-down. Lane 2 was loaded with whole cell lysate from the same experimental batch as input. (**E**) Visual summary of DYRK1A-Wnt protein-protein interactions identified by the Jerome LeJeune foundation interPP project (Y2H screen, double coloured lines), literature sources (solid black line) and experimental evidence (dotted black line). Red and blue lines represent differential degrees of confidence in interaction strength. Red circled proteins were selected for further STRING network investigation as first shell. (**F**) Extended DYRK1A network from proteins selected in (**E**) adding 10 direct interactors for each node and a further 10 secondary interactors, demonstrating DYRK1A may be closely associated with protein-protein interactions involved in Wnt signalling. Major pathway components such as β-catenin were automatically integrated in the network by STRING.

To investigate DYRK1A as a candidate Wnt signalling modulator, we assessed whether this protein was able to physically interact with components of the cascade. We first probed the entire DYRK family of genes (*DYRK1A/B*, *DYRK2-4*) for association with the Wnt pathway (Fig. S3), employing the STRING protein interaction database, v10.0^58^. For all DYRK family members, a significant association was detected with the gene ontology (GO) and KEGG terms ‘Wnt signalling pathway’. We then consulted a database of protein-protein interaction networks in DS, generated via Yeast Two-hybrid (YTH) screening. We searched the database for DYRK1A interactors with known functional relevance to Wnt signalling. In yeast, DYRK1A interacted with the Wnt inhibitor DKK3 at amino acids 589–763 (Fig. S4), a sequence reportedly important for its nuclear localisation^59^. DYRK1A was also found to interact with Lipoprotein receptor-related protein (LRP) 1/1B/4, Wnt inhibitory factor 1 (WIF1), Wnt3 and α-catenin (Summarised in Fig. 4D).

We then employed co-immunoprecipitation (coIP) in HEK293 cells validating the DYRK1A-DKK3 interaction (Fig. 4A–D). We also found that DYRK1A precipitated with the key Wnt transducer disheveled-1 (DVL1), but not β-catenin (Fig. 4B,C). Importantly, we consistently observed DYRK1A interactions with DVL1 and DKK3 via overexpression (Fig. 4A–C) as well as endogenously (Fig. 4D). The latter finding suggests the physiological occurrence of these protein interactions. To contextualise them further, we employed the Cytοscape software to build a network of potentially relevant DYRK1A-Wnt interactions and pathways. We combined the most reliable DYRK1A partners from YTH, coIP and literature data (Fig. 4E, red circles) into a single network enriched via STRING (Fig. 4F). Interestingly, we found that some of the network nodes were altered in humans DS and Tc1 mouse hippocampi, as shown in our biochemical and transcriptomic studies (Fig. 4F, blue circles). Overall these data suggest DYRK1A might be involved in Wnt signalling protein interaction networks, and may thus be able to influence Wnt function.

### DYRK1A is a bimodal Wnt signalling regulator

**Figure 5:**
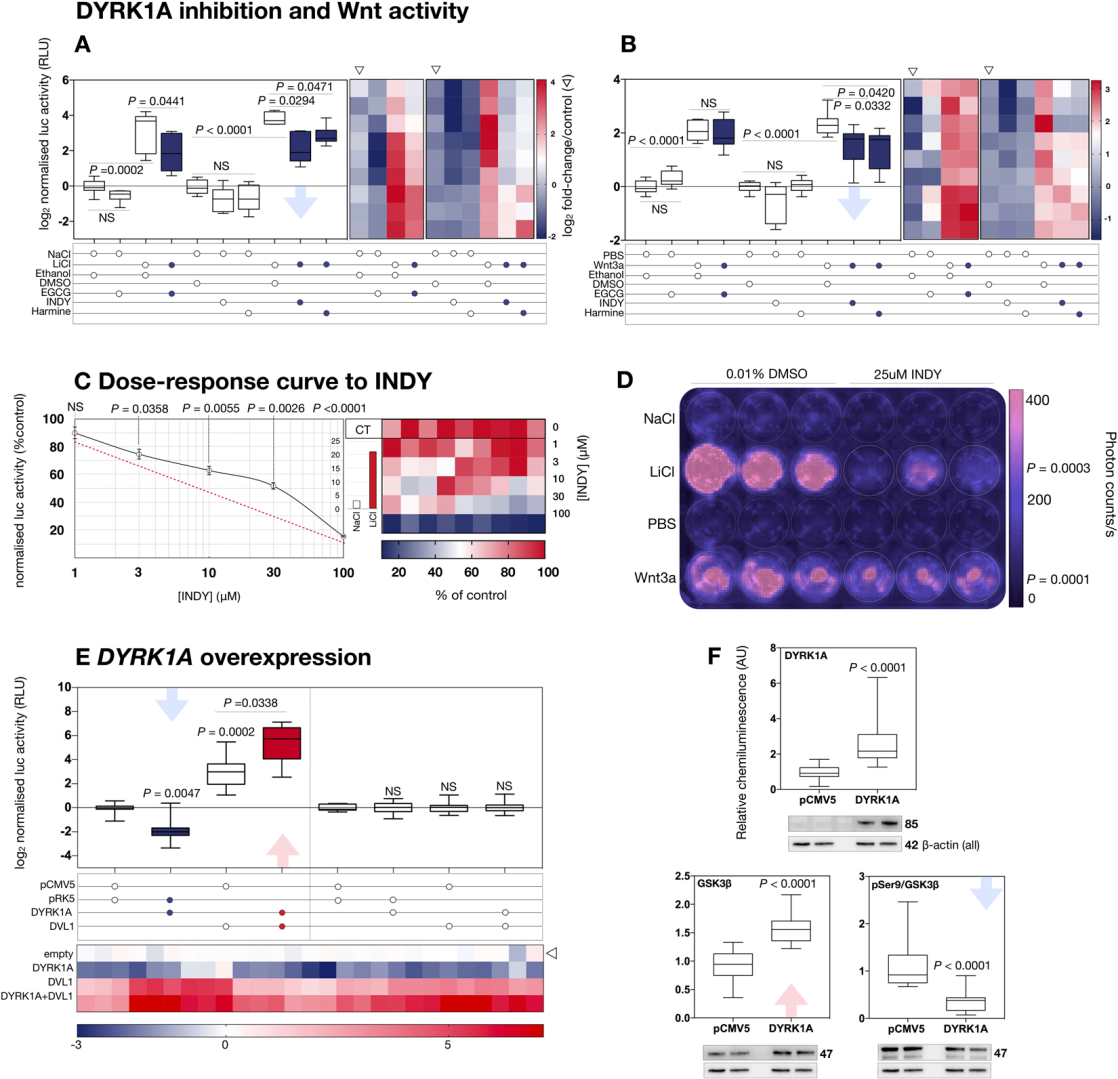
DYRK1A is a bimodal canonical Wnt signalling modulator in a human cell line. (**A**) DYRK1A inhibition reduces levels of LiCl-induced canonical Wnt signalling activity quantified via TOPflash luciferase assay. SH-SY5Y cells stably expressing the TCF-LEF luciferase reporter (n = 9) were treated with 40 mM LiCl or NaCl control for 5 hours with or without 25 μM EGCG, 25 μM INDY or 10 μM Harmine. 0.1% ethanol and 0.1% DMSO were employed as negative control treatments for EGCG and INDY/Harmine, respectively. All inhibitors significantly reduced activation (blue bars). Heat map represents log_2_fold changes/NaCl alone for individual luciferase-expressing cultures. (**B**) same as (**A**) but with 50 ng/ml Wnt3a stimulation. All inhibitors but EGCG significantly reduced activation (blue bars). (**C**) The inhibitory effect of INDY on LiCl-induced activation is dose-dependent. Doses of 1–100 μM INDY (n = 9) were administered for 5 hours and luciferase activity was plotted as percentage of control treatment. Linearity was observed (*P* < 0.0001, R^2^ = 0.64, f(x) = −0.7× + 83). Heat map represents values normalised to LiCl +0.1% DMSO for individual cultures. (**D**) Effect of INDY in live, non-lysed stable SH-SY5Y cells (*n* = 9). Samples were treated as in (**A**,**B**) with or without 25 μM INDY, and imaged employing the IVIS system. (**E**) Effects of DYRK1A expression in the SH-SY5Y line. Cells (n = 27) were co-transfected with 0.25 μg/ml HA-DYRK1A, DVL1, both, or empty vector controls for 24 hrs. 0.25 μg/ml TOPflash and 0.025 μg/ml constitutively active renilla luciferase constructs were employed as reporters, with signal quantified as TOPflash/Renilla ratio. All values expressed as log_2_fold relative to empty vector transfection. Adjacent graph demonstrates same experiment employing mutant reporter construct FOPflash-luciferase. Heat map represent values for individual luciferase-expressing cultures. (**F**) Immunoblot demonstrating DYRK1A expression is sufficient to enhance total amounts of GSK3β protein and alter its phosphorylation status at Ser9. SH-SY5Y cells (n = 12) were transfected with 0.5 μg/ml HA-DYRK1A for 24 hrs. Total levels of GSK3β were enhanced, and phosphorylation at Ser9, as expressed by ratio to total protein, was significantly reduced.

We next determined whether DYRK1A could modulate the transcriptional activity of β-catenin in human cells. First, the effect of DYRK1A kinase inhibition on LiCl or Wnt3a-driven Wnt signalling activity was investigated in a neuroblastoma (SH-SY5Y) cell line stably expressing a Wnt signalling luciferase reporter (Fig. 5A,B). We employed three DYRK1A inhibitors, EGCG^60^, INDY^61^ and Harmine^62^, at previously published doses to achieve target-specific inhibition (25 μM EGCG/INDY, 10 μM Harmine). Overall, DYRK1A inhibition reduced *active* but not *basal* Wnt signalling following stimulation with LiCl or Wnt3a (Fig. 5A,B, blue; Fig. 5D). As expected, no dose-dependence was observed for INDY effects on *basal* Wnt levels (Fig. S5), whereas the effect of INDY on *active* signalling was dose-dependent (Fig. 5C, red dotted line) within a target-specific range^61^. Interestingly, Wnt-activating Lithium treatment, here influenced by DYRK1A inhibition, has been previously found to rescue cognitive defects and synaptic plasticity in the Ts65Dn mouse model of DS^63^.

Next, we investigated whether canonical Wnt signalling activity could be affected by DYRK1A overexpression. Surprisingly given the inhibition data, we found *basal* activity was potently downregulated by DYRK1A overexpression, with a significant reduction to nearly undetectable levels (Fig. 5E, blue). However, DYRK1A exerted a diametrically opposite effect on *active* signalling, in accordance with the kinase inhibitor experiments. When co-expressed with DVL1, the resulting luciferase-reported signal was enhanced approximately eight-fold compared to DVL1 alone (Fig. 5E, red). Given the inhibitory effect on basal signalling activity, we tested whether DYRK1A overexpression could affect protein levels of GSK3β, a principal intracellular inhibitor of active β-catenin. We identified a significant increase in total GSK3β levels (Fig. 5F, red) along with reduced phosphorylation of the inhibitory Ser 9 residue (pSer9, Fig. 5F, blue) in accordance with decreased canonical Wnt signaling activity.

In summary, increases in DYRK1A result in reduction of *basal* Wnt signalling activity but further increases *active* Wnt signalling substantially. The activation-dependence of the latter effect is supported by the kinase inhibitor data, as DYRK1A kinase inhibition reduces active Wnt signalling. In our system, however, DYRK1A kinase inhibition has no measurable effect on basal Wnt signalling activity. These data overall suggest the presence of newly identified, bimodal Wnt signalling regulation by DYRK1A.

### Wnt signalling activation induces cytoplasmic redistribution of nuclear DYRK1A

**Figure 6:**
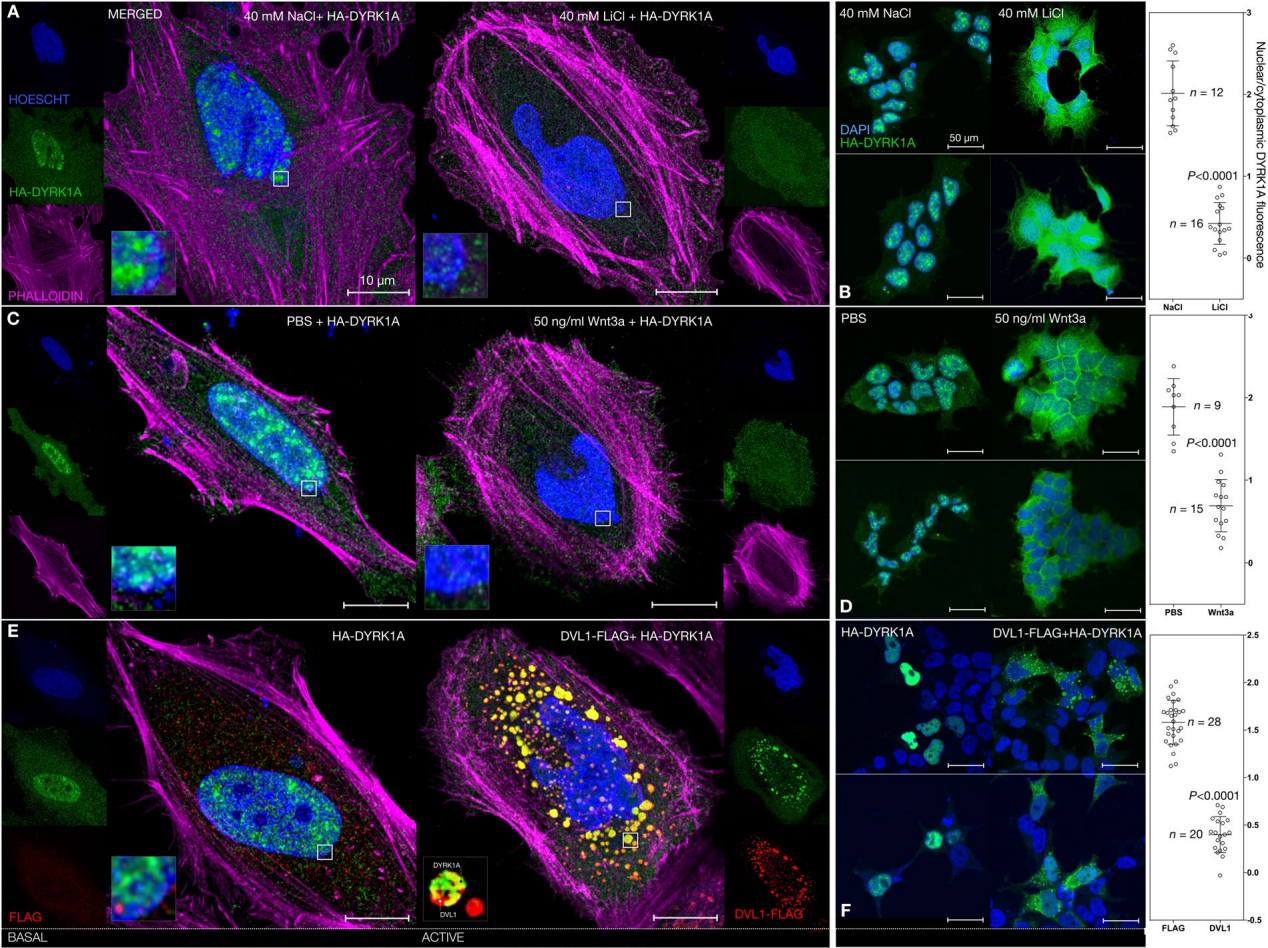
Subcellular localisation of DYRK1A is affected by Wnt signalling activation. (**A**) Airyscan microscopy of 5-hour 40 mM NaCl (left) or LiCl (right) treatment in HeLa cells. DYRK1A is pseudocolored in green, DAPI (nuclear stain) in blue, and phalloidin (F-actin stain) in magenta (applies to all panels). DYRK1A localised to the nucleus when expressed alone (left), but redistributed to the cytoplasm in the presence of wnt activation. (**B**) Nuclear/cytoplasmic fluorescence ratio quantified from the same conditions as in (**A**) but in HEK-293 cells, demonstrating a significant reduction in nuclear localisation. (**C**) Same as (**A**) but employing 5-hour PBS as basal control and 50 ng/ml Wnt3a to achieve Wnt activation. Decreased DYRK1A nuclear fluorescence was observed (right). (**D**) Same as (**B**) but for experiment in (**C**). A significant reduction in the nuclear/cytoplasmic ratio was observed. (**E**) Same as (**A**,**C**) but employing 24-hour overexpressed 0.5 μg/ml HA-DYRK1A ± DVL1-FLAG (red) with prominent cytoplasmic co-localisation in the presence of DVL1. (**F**) Same (**B**,**D**) but for experiment in (**E**). A significant reduction in the nuclear/cytoplasmic ratio was observed.

**Figure 7:**
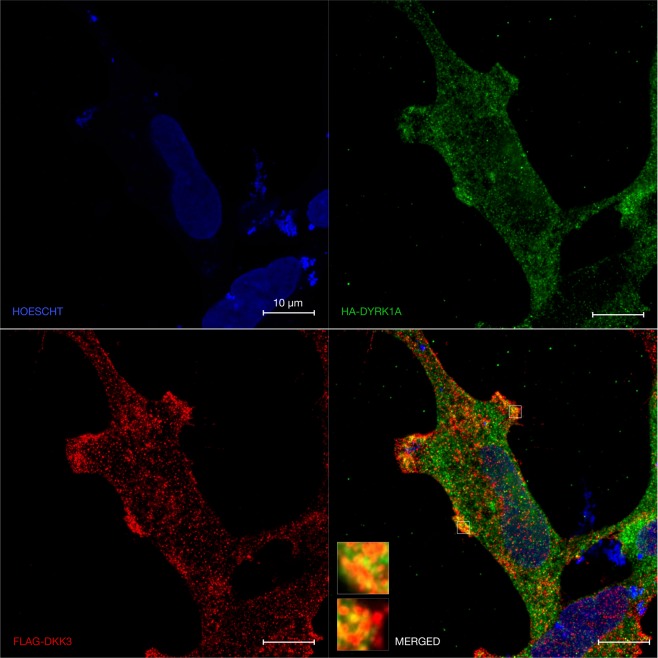
Subcellular localisation of DYRK1A and DKK3. 24-hour overexpression of 0.5 μg/ml HA-DYRK1A (green) ± DKK3 (red) showed cytoplasmic distribution for both proteins with some co-localisation near the plasma membrane.

Given the observed bimodal Wnt effects of DYRK1A, we hypothesised that the subcellular localisation of this kinase may be modified by Wnt activation states. A shift in distribution and availability of DYRK1A pools may account for its differential regulation of Wnt signalling activity. DYRK1A localises prominently to the nucleus but also the cytoplasm^27,59,64,65^. In HeLa and HEK293 cells, overexpressed DYRK1A localisation was predominantly nuclear under basal Wnt signalling activity conditions (Fig. 6A–F left, green). Upon Wnt signalling activation however, we observed a statistically significant redistribution of the DYRK1A signal out of the nucleus, with the kinase displaying a more diffuse cytoplasmic localisation pattern (Fig. 6A–F right, green). Upon 24-hour DVL1 overexpression, (Fig. 6E, right, red) DVL1 displayed a distinctive vesicle-like distribution, currently ascribed to liquid-liquid phase transition^66,67^. Remarkably, DYRK1A almost completely redistributed to the cytoplasm, showing substantial co-localisation with DVL1 (Fig. 6E, right, yellow). This finding is in accordance with the observed DYRK1A-DVL1 protein interaction (Fig. 4B), and might constitute the basis for enhanced Wnt signalling activity upon overexpression of DYRK1A (Fig. 5E). We also sought to initially determine the subcellular localisation, if any, of our reported DKK3-DYRK1A interaction (Fig. 7). When co-expressed with DKK3 in HEK239 cells, DYRK1A displayed prominent cytoplasmic distribution (Fig. 7 top right, green), whilst DKK3 accumulated towards the cell membrane (Fig. 7 bottom left, red). Within this site, several areas of increased signal co-localisation with DYRK1A were observed (Fig. 7 bottom right and detail boxes, yellow). Whilst this interaction remains to be further characterised, our preliminary data suggest that DKK3 and DYRK1A might functionally interact at the cell membrane. Given the above discussed interaction with DVL1, which our findings indicate may result in Wnt signalling modulation, we suggest that DKK3 might further modulate this interaction.

**Figure 8:**
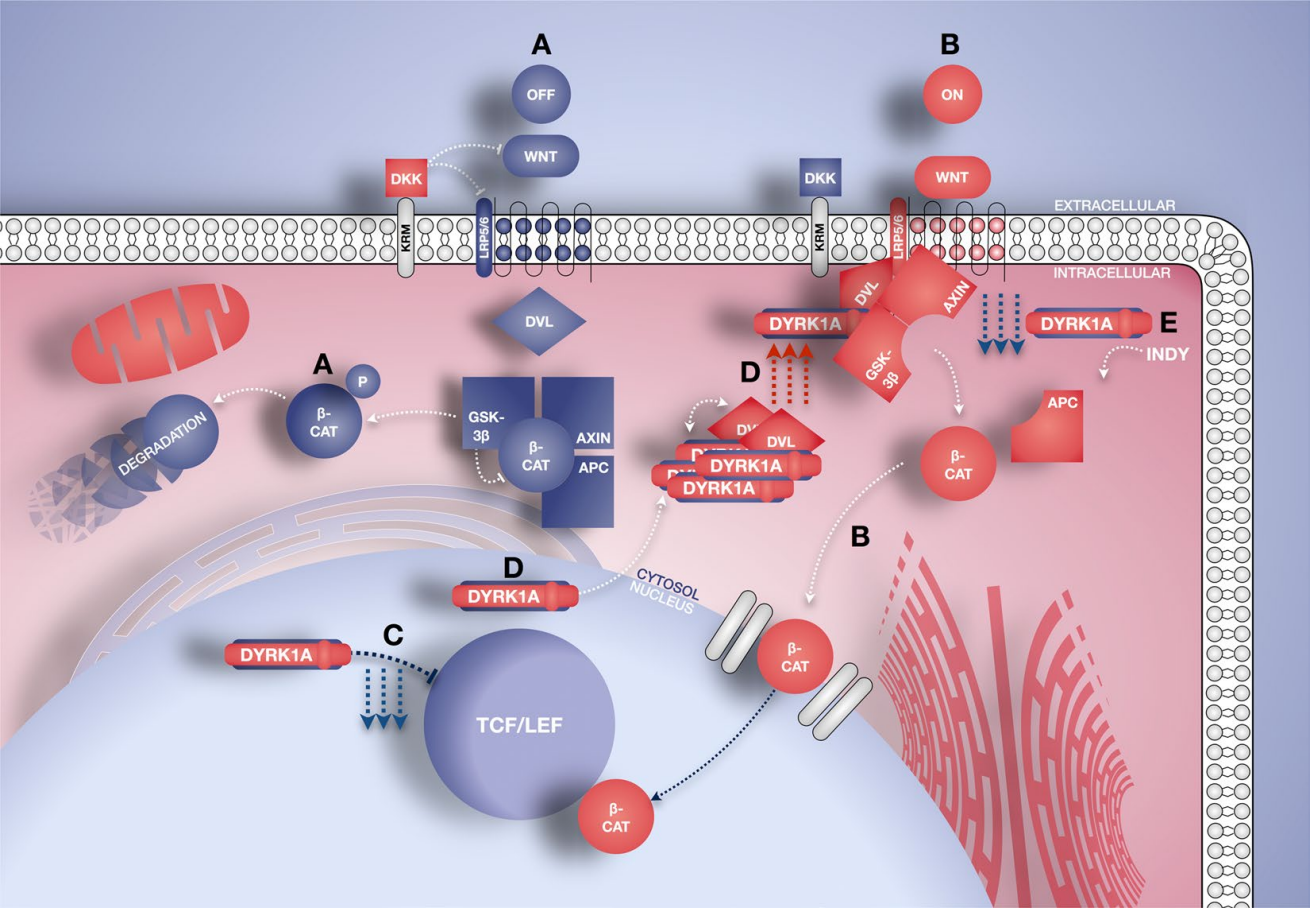
Diagrammatic model summarising the proposed bimodal Wnt signalling regulation by DYRK1A, as reported in Figs 4–6. (**A**) Basal/Inactive Wnt signalling conditions, whereby nuclear translocation of β-catenin is prevented via its proteasomal degradation, mediated by the destruction complex. (**B**) Active Wnt signalling, whereby Frizzled receptor stimulation by ligands results in membrane recruitment of the destruction complex, cytosolic release and nuclear translocation of ‘free’ β-catenin followed by TCF/LEF binding and transcriptional activation of Wnt target genes. (**C**) *DYRK1A* overexpression alone results in significant downregulation of Wnt signalling activity (Fig. 5E, blue) along with prominent nuclear localisation of the DYRK1A protein (Fig. 6A–F, left green). (**D**) *DYRK1A-DVL1* co-expression (ie under active Wnt signalling conditions) significantly enhances Wnt signalling activation levels (Fig. 5E, red) and leads to cytoplasmic accumulation of DYRK1A (Fig. 6E,F, right green), likely mediated by direct interaction with DVL1 (Figs 4B,D and 6E, right yellow). This DYRK1A redistribution pattern is also observed following Wnt stimulation with LiCl or Wnt3a (Fig. 6A–D, right green). (**E**) Consistently, DYRK1A inhibition results in decreased levels of active Wnt signalling following LiCl or Wnt3a treatment, relative to pathway stimulation alone (Fig. 5A–D). **APC** - Adenomatous polyposis coli; **β-cat** - β-catenin; **DKK** - Dickkopf-related protein; **DVL** - Disheveled 1; **GSK-3β** - Glycogen synthase kinase 3β; **KRM** - Kremen co-receptor; **LRP5/6** - Low-density lipoprotein receptor-related protein 5/6; **P** - Phosphorylation; **TCF/LEF** - T-cell factor/lymphoid enhancer factor.

Overall, these data further support the notion of a strong, bilateral functional relationship between DYRK1A and Wnt signalling, whereby DYRK1A modulates pathway activity, and the cascade itself regulates DYRK1A localisation (Fig. 8).

## Discussion

Here, we showed for the first time dysfunction of Wnt/β-catenin signalling in human DS (Fig. 1). This key developmental pathway is well placed to underlie many of the wide-ranging features of DS, and particularly the neurodegeneration found in AD-DS^18–24^. Our data adds further evidence that the DS transcriptome is deregulated beyond Hsa21 genes (Figs 2 and 3 and^6,68,69^ and supports recent findings of overall cell signalling defects in DS^70,71^. We hereby propose that dysfunction of the Wnt signal transduction pathway contributes to DS phenotypes. Chronic Wnt dysregulation, due to overexpression of modulators on Hsa21, in particular *DYRK1A*, may effectively extend the dosage imbalance effects of one gene to a high number of secondary target genes.

This conclusion is supported by our combined genetic and biochemical analyses of human DS and Tc1 mouse hippocampal samples (Figs 1–3). Wnt signalling activity was consistently downregulated in the hippocampus of Tc1 mice and human DS individuals. In the former, downregulation of Wnt signalling was suggested by transcriptional profiles as well as reduced active β-catenin protein levels. Given the premature onset of AD dementia in human DS^50^, whereby pathology manifests as early as the third decade of life, initial Wnt investigation in the Tc1 model focused on young adult mice. Our findings thus suggest that hippocampal Wnt signalling reduction may represent an early pathological process, potentially underlying the premature development of AD in human DS. It would nevertheless be of interest to determine whether, in the Tc1 mouse, similar changes might also be present at a later time point, as already observed in human DS hippocampi. This should be addressed in future studies.

Indeed, current knowledge of Wnt signalling in neuronal function indicates that downregulation of the pathway is likely to contribute to neurodegenerative processes, especially during adulthood^18–24^. Further reflecting underlying abnormalities in Wnt signalling, we detected changes in DKK3 levels in Tc1 and human DS hippocampi. DKK3 is a member of the Wnt inhibitor Dickkopf family, which is involved in hippocampal development and AD^20,72^. Consistent with our data, DKK3 is reportedly elevated in CSF and plasma of sporadic AD patients^73^. It is presently unclear why the DKK3 alterations differ between the Tc1 mouse and humans. This is possibly due to late-stage overall dysfunction of the Wnt secretory machinery in AD-DS, and further investigation is required.

We suspect, however, that Wnt signalling might not necessarily be ubiquitously downregulated in DS. The complexity of Wnt function in development, health and disease generally supports the possibility of multi-directional alterations in DS^9,74,75^, although this remains to be determined. By contrast, mice with duplicated Hsa21 syntenic regions on Mmu10 or 17 presented no overall effect on active β-catenin (Fig. S2). Therefore, we assumed that the overexpression of genes located on the Hsa21 syntenic region on Mmu16 affects canonical Wnt signalling in the hippocampus. This led to our hypothesis of DYRK1A as a candidate Wnt modulator in DS, given its known functional interaction with Wnt components^28,31,35^ and important contribution to DS and AD^37–45^.

DYRK1A was able to regulate total and pSer9 GSK3β levels in SH-SY5Y cells, leading to Wnt signal inhibition under basal conditions (Fig. 5F). This is perhaps not surprising, given the known priming relationship between the two kinases. Evidence suggests DYRK1A may employ priming phosphorylation to target substrates for further modification by GSK3β and subsequent degradation, independently of Wnt function^28,76–78^. A similar mechanism might however allow DYRK1A to direct GSK3β phosphorylation to Wnt signalling components. The presence of several DYRK1A interactions with Wnt-associated proteins, most importantly DVL1 and DKK3 (Figs 4, S3 and 4) suggests multiple access routes for DYRK1A modulation of the Wnt pathway. It is particularly interesting that DKK3, which we found to be altered in the DS hippocampus, interacts with DYRK1A via its nuclear speckle-localising sequence^59^. This suggests potential regulation of DYRK1A localisation by DKK3, a possibility which warrants further study. The bimodal effects of DYRK1A on Wnt signalling activity support its modulatory role further (Figs 5A–E, 7). Overall, DYRK1A enhanced active Wnt signalling while reducing basal activity levels. Consistently with this idea, we showed subcellular re-localisation of DYRK1A from the nucleus^27,59^ to the cytoplasm upon Wnt signalling activation (Figs 6 and S6). This suggests that bimodal Wnt regulation by DYRK1A may be dependent upon its subcellular distribution and resulting substrate preference. Nuclear DYRK1A may thus suppress basal Wnt signalling activation by a transcriptional process^27^. By contrast cytoplasmic DYRK1A, possibly working in synergy with its interaction partner DVL1, increases active Wnt signalling further. This novel Wnt enhancing property of DYRK1A is supported by recent findings in pancreatic β-cells^79^. It is possible that these novel properties of DYRK1A may account for, or at least contribute to, the beneficial effects of Lithium treatment in the Ts65Dn DS mouse model reported by Contestabile *et al*.^63^. The notion of a functional Wnt-DYRK1A relationship is also supported by our preliminary identification of a physical interaction of this kinase with DKK3 (Figs 4, S3 and 4), which might occur intracellularly at the plasma membrane (Fig. 7). Whilst we do acknowledge that DKK Wnt antagonists are mostly known as secreted proteins^72^, evidence exists suggesting DKK3 might also possess intracellular functions^80^. Thus we preliminary propose that, DKK3 might further contribute to bimodal Wnt-DYRK1A modulation by interacting with the latter kinase at the cell membrane.

The consequence of this dualistic effect in DS might be a complex spatiotemporal alteration in Wnt target gene expression throughout life (Fig. 8). Given that the default level of *DYRK1A* expression is chronically elevated in this condition, Wnt signalling might be proportionally affected as a consequence, in a direction determined by local activation states. This implies that tissue types with low basal Wnt signalling levels, such as adult hippocampal neurons, may suffer from further downregulation, as observed in Tc1 mice and DS individuals. Indeed, under-activation of Wnt signalling in the adult hippocampus results in synaptic loss and neurodegeneration^20,81,82^ and is expected to disrupt key mechanisms such as neurogenesis^83,84^ and long-term potentiation^85^. Conversely, rapidly proliferating tissues such as fibroblasts may undergo aberrant Wnt over-activation. It is well established that enhanced Wnt activity significantly correlates with development of several cancer types^9,74,75,86^. Thus, Wnt dysfunction might potentially contribute to the known differential cancer susceptibility in DS, such as the enhanced risk of leukemias and reduced incidence of solid tumours^87^. High basal Wnt levels in proliferative haematopoietic cells might, for example, be exacerbated by *DYRK1A* overexpression, which does indeed play a role in DS-related leukemias^88^. This intriguing possibility, however, remains to be investigated.

## Conclusions

Overall, the key implication of our findings is that Wnt signalling regulation by DYRK1A may be established as a novel target for therapeutic development in DS neuropathology and beyond. Such a strategy might entail a dual approach: (1) Targeting aberrant DYRK1A activity in particularly Wnt-susceptible tissue types such as the adult hippocampus. (2) Directly targeting under/over-activation of Wnt signalling and target genes, depending on tissue-specific directional changes. Both approaches are particularly attractive, chiefly because Wnt signalling and DYRK1A have been heavily investigated therapeutically. In the context of cancer, a high number of pharmaceutical agents which target Wnt signalling on multiple levels already exists^14,89,90^. Stimulation of neuronal Wnt signalling, on the other hand, is currently viewed as a promising strategy in AD and other neurodegenerative diseases^18,63,91,92^. Similarly, employment of DYRK1A inhibitors to target cognitive deficits in DS and β-cell dysfunction in diabetes is a rapidly developing field^27,45,60,62,93,94^. A variety of therapeutic strategies can be envisioned at multiple developmental stages. Administration of a combined Wnt-DYRK1A normalising therapy *in utero* might prove critical in tackling developmental abnormalities in DS. Likewise, Wnt signalling stimulation may be beneficial in later life, especially in the context of AD-DS. Thus, eventual clinical translation of our findings might open avenues for treatment strategies in DS, aimed at normalising Wnt signalling function throughout life. We hope that future research efforts will expand on our proposed model, in order to tackle this condition and improve the quality of life for those affected by it.

## Methods

### Study design

#### Sample size

Selected based on sample/tissue availability following standard procedures for each technique, in order to generate statistically significant results, see relevant sections of materials and methods.

#### Data inclusion/exclusion criteria

All data collected for each experiment presented were included.

#### Outliers

No outliers were excluded, all data are presented as box plots including minimum and maximum values.

#### Replicates

For all mouse and human DS immunoblotting experiments, samples were quantified in duplicate and averaged, reported *n* for these experiments represent averaged duplicates. For example, *n* = *8* Tc1 hippocampal samples indicates analysis of 16 total samples (including 8 WT controls) measured twice (total of 32 values quantified) in separate gels and averaged. For all cell-based experiments, a minimum of three independent repeats was performed, with variable sample sizes reported in the main text and legends. In this case, *n* represents all individual replicates added together. For further information on the above and all other analyses conducted see Supplementary Tables S4 and 5 and relevant sections of materials and methods.

Pre-specified hypotheses:

1. Wnt signalling dysregulation may be associated with gene expression and/or protein profiles of DS mouse models and humans.
2. One or more Hsa21-encoded protein may functionally regulate Wnt/β-catenin signalling, particularly:

A. Interact with Wnt components
B. Affect luciferase-reported Wnt signalling activity

Hypotheses suggested after initiation of the data analyses:

1. The Wnt phenotypes observed in DS models and humans are likely to be mediated by Mmu16 Hsa21 orthologs.
2. Lack of *APP* expression in the Tc1 mouse indicates *DYRK1A* as a more likely Wnt modulator in our current system, and this gene was thus tested for pre-specified hypotheses 2A,B
3. Wnt signalling activation states may affect subcellular distribution of DYRK1A,

#### Research subjects or units of investigation

Tc1 mouse hippocampal RNA; Tc1, Dp(17)1Yey and Dp(10)1Yey hippocampal samples, post-mortem hippocampal samples of DS patients; Hybrigenics-generated Y2H interaction database from the Inter-PP project (Jerome LeJeune Foundation); SH-SY5Y, HEK-293 and HeLa cell cultures.

#### Experimental design

Controlled laboratory experiments, see relevant sections of main text and materials and methods for types of measurements made.

### Statistical analysis and graphs

RNAseq data was analysed by Deseq. All *P* values reported were adjusted for false discovery rate (FDR). All IPA-generated *P* values reported for pathway, upstream regulator, and disease and function analyses were produced by Fisher’s exact test and thresholded at *P* < 0.05. STRING protein interaction networks for the DYRK family were analysed by STRING, correcting for FDR. All other statistical analyses were performed in GraphPad Prism 07a. For human and mouse biochemistry and qPCR reported *P* values were calculated by two-tailed Mann-Whitney *U* test, due to the non-Gaussian nature of the datasets. DYRK1A expression experiments and immunocytochemistry were also analysed with the same test. For all Luciferase experiments, the data were analysed by Kruskal-Wallis test with post-hoc correction for multiple comparisons by two-stage linear step-up procedure of Benjamini, Krieger and Yekutieli, due to the non-Gaussian nature of the datasets. All *P* values there reported represent FDR-adjusted *q* values. For dose-response curve experiments (Fig. 5C), treatment groups/doses were matched by individual experimental repeat and analysed by Friedman test with the same correction as above. Linear regression was calculated on % of maximum (LiCl + DMSO) values, throughout the entire dose range of 1–100 μM. See Supplementary Tables S4 and 5 for additional info on all statistical tests performed. All heat maps and box plots were created in GraphPad Prism 07. In all cases, box span represents first to third quartiles, bands represent the median of each sample group, and whiskers represent minimum/maximum values. Throughout this study, relative ‘increases’ were colour coded as red, and ‘decreases’ as blue in a combination of hues safe for all types of colour vision deficiency. See relevant sections of materials and methods for additional details on quantification and processing methods employed.

### Expression constructs

pCMV5-HA-DYRK1A and pCS2-DKK3-FLAG were obtained from MRC PPU Reagents, while pCDNA3.1-FLAG-β-catenin was a gift from Eric Fearon (Addgene plasmid #16828). pRK5-FLAG-DVL1 was generated in-house as described previously^95^. M50 Super 8X TOPflash and M51 Super 8X FOPflash were gifts from Randall Moon (Addgene plasmids #12456 and #12457)^96^. pTK-Renilla (Renilla) was purchased from Promega. All plasmid were verified by DNA sequencing at MRC PPU Sequencing Services (Dundee, Scotland, UK).

#### Animal husbandry, welfare, and tissue sampling

Tc(Hsa21)1TybEmcf (Tc1), Dp(17Abcg1-Rrp1b)1Yey (Dp(17)1Yey), and Dp(10Prmt2-Pdxk)1Yey (Dp(10)1Yey), mice^47,54^ were bred at the Francis Crick Institute in specific pathogen free conditions, in a controlled environment and in accordance with the MRC Responsibility in the Use of Animals for Medical Research (1993) guidelines. Tc1 mice were maintained by crossing to (C57BL/6J × 129S8)F1 mice; all other strains were maintained by crossing with C57BL/6J mice. All animals were euthanised by cervical dislocation in accordance with the Animals (Scientific Procedures) Act 1986 and European Directive 2010/63/EU. For immunoblotting experiments, brains were collected from 160 ± 28 days days old male Tc1 mice and 131 ± 7 days old male Dp(17)1Yey/Dp(10)1Yey mice, washed in phosphate-buffered saline (PBS) and dissected into bilateral cortical, hippocampal and brainstem fractions. Samples were then snap-frozen in liquid nitrogen and stored at −80 °C. Prior to each experiment, all mouse hippocampal samples were homogenised via mechanical disruption, at 4 °C, in 500 μl brain lysis buffer [50 mM Tris, pH 7.5, 150 mM NaCl, 5 mM MgCl_2_ and 1% (v/v) NP-40, 1x complete protease inhibitor cocktail (Roche), 1x Halt phosphatase inhibitor cocktail (Thermo Scientific™)]. Each lysate was clarified at 4 °C by centrifugation at 20,000 *g* and denatured via addition of 10x sample reducing agent and 4x LDS sample loading buffer (Thermo Scientific™) and heating at 99 °C for 5 min. All samples were assayed for protein concentration by bicinchoninic acid (BCA) assay (Thermo Scientific™).

### Cell maintenance

For luciferase, coIP experiments and imaging experiments, SH-SY5Y and HEK293 cells were grown to 10^6-7^ cells/ml (counted with Muse® count and viability kit) and maintained at 37 °C and 5% CO_2_ in 10 ml Dulbecco’s modified Eagle’s medium (DMEM) enriched with 10% (v/v) foetal bovine serum (FBS), 100 U/ml penicillin G, 100 μg/ml streptomycin and 2 mM glutamine. For immunocytochemistry experiments, HeLa cells (ATCC) were grown in phenol red-free Dulbecco’s Modified Eagle Medium (DMEM) supplemented with 10% (v/v) FBS (Corning), 2 mM L-glutamine, 100 U/ml penicillin and 100 μg/ml streptomycin at 37 °C and 5% CO_2_.

### Generation of SH-SY5Y cells stably expressing the the TCF/LEF-Luciferase reporter (Stable TOPflash)

Stable TOPflash SHSY5Y cells (Fig. 5A–D) were made in a two-step process. Firstly, the TCF/LEF-responsive promoter and luciferase reporter from M50 Super 8x TOPflash^96^ was cloned into a plasmid with suitable antibiotic resistance for selection in mammalian cells. To this end, the CMV promoter was removed from pcDNA3 by restriction digestion with BglII and NotI, and a NotI-BamHI fragment from M50 Super 8X TOPflash ligated into these sites. The responsiveness of the resultant plasmid to transient transfection with DVL1 was confirmed by luciferase assay and found to be approximately ~25% that of M50 Super 8x TOPflash. Secondly, this plasmid was stably transfected into SH-SY5Y cells. The plasmid was linearised by PvuI digestion and transfected into SH-SY5Y cells, with stably transfected cells selected for resistance to G418 (800 μg/ml) following standard procedures. Twelve clonal cell lines were established and compared for basal luciferase expression and fold-induction in response to 30 mM LiCl treatment. Clone #3 was chosen for experiments performed in this study, as the cell line displaying the greatest sensitivity to Wnt pathway activation.

### Transfection

Cells were treated with 3:1 FuGENE® HD transfection Reagent (Promega) for 24 hrs to achieve transient gene expression. In all luciferase experiments, 0.25 μg/ml DNA per construct/condition were transfected. In *DYRK1A* expression experiments and coIPs, 0.5 μg/ml DNA per construct/condition were transferted. Cells were subsequently lysed with in 1 ml cell/brain lysis buffer [150 mM NaCl, 50 mM Tris (pH 7.5), 2 mM EDTA (pH 8), 1% (v/v) Triton X-100, 1x complete protease inhibitor cocktail and 1X Halt phosphatase inhibitor cocktail]. Lysate clearing and denaturing was achieved in the same conditions as above.

### Post-mortem Human DS samples

All post-mortem human brain samples were processed in accordance with the Human Tissue Act 2004 and directives from the Human Tissue Authority (UK). The study was reviewed and approved by NHS Research Ethics committee, London-Queen Square. Samples were provided, anonymised, by the Newcastle brain bank, from 55 (±11) and 56 (±12) years old DS/AD patients and age-matched euploid controls, respectively (Fig. 1A and Table S1). All patients had granted full research consent. 10 mg of frozen tissue from hippocampal and cortical samples was excised in a sterile environment and immediately triturated with a plastic pestle in 100 μl brain lysis buffer. All samples were then cleared and denatured as described above, and protein concentration was measured as described.

### Pharmacological treatment of cells

Wnt signalling activation was achieved by treatment with either 40 mM LiCl or 50 ng/ml Wnt3a (R&D) for 5 hrs. 40 mM NaCl and 0.1% PBS were employed as control treatments, respectively. The DYRK1A inhibitors Epigallocatechin-3-gallate EGCG (Sigma-Aldrich), INDY (Tocris) and harmine (Tocris) were prepared according to manufacturer’s instructions and administered at previously published doses of 25 μM (EGCG), 1–100 μM (INDY) and 10 μM (harmine) for 5 hr. 0.1% Ethanol or 0.1% DMSO were employed as controls for EGCG and INDY/harmine, respectively.

### Western blotting

Approximately 10 μg protein from all mouse, human cell line and human post-mortem samples were loaded into 10 or 20-well 4–12% (*w/v*) BisTris pre-cast gels (Thermo Scientific™) and subsequently transferred onto polyvinylidine fluoride (PVDF) membranes (Biorad). Membranes were blocked for 1 hr in Tris-buffered saline, 0.1% Tween 20 (TBS-T) with 5% (*w/v*) non-fat dry milk. All primary antibodies were administered overnight in blocking buffer, at 4 °C. Following at least three washes in TBS-T, membranes were treated with HRP-conjugated secondary antibodies for 1 hr at room temperature and washed again. All primary and secondary antibodies were employed at 1:2000 dilution, except for the β-actin antibody used 1:5000 (Table S6). Protein bands were visualised with SuperSignal West Pico/Femto Chemiluminescent HRP Substrate (Thermo Scientific™). Images were acquired with a SynGene GeneGnome Imaging system with varying exposure times from 10 sec to 1 min depending on signal strength.

### Luciferase assays

All luciferase assays were carried out in 6-well plates and as previously described^97^. For experiments depicted in Fig. 5A–C,E, a Dual Luciferase Reporter assay kit (Promega) was employed either in stable TOPflash cells (Fig. 5A–C) or via transient expression of 0.25 and 0.025 μg/ml of TOP/FOPFLASH and renilla luciferase constructs in unmodified SH-SY5Y, respectively (Fig. 5E). For the latter, 0.25 μg/ml of empty vector and/or HA-DYRK1A and FLAG-DVL1 were also co-transfected as described above. Transient expression of DYRK1A was assessed against basal and DVL1-driven canonical Wnt signalling activity at 24 hours. For results in Fig. 5D, luciferase activity was measured in live cells from the TOPflash stable line, by enriching DMEM with 150 ug/mL D-luciferin (Systems Biosciences) and employing the IVIS system (Perkin-Elmer) to image plates 20 min post-treatment.

### Co-immunoprecipitation

HEK293 cells were co-transfected with 0.5 μg/ml HA-DYRK1A and 0.5 μg/ml FLAG-DVL1/β-catenin-FLAG/DKK3-FLAG or relative empty controls and lysed as described above. Prior to denaturing, 40 μl of anti-FLAG M2 affinity gel (Sigma) were added to 1 ml of each lysate. Following 1 hr of rotational incubation at 4 °C, the gel was washed five times by centrifugation and resuspension in cell lysis buffer. 150 ng 3xFLAG peptide (Sigma) were used to elute the fusion proteins. Eluates were denatured as described above. Endogenous coIPs were also performed in HEK293 cells under basal conditions, employing Protein A Dynabeads® (Thermofisher) conjugated to a DVL1 or DYRK1A antibody, following protocols supplied by the manufacturer. Each coIP was performed independently at least three times.

### RNAseq

Total male Tc1 hippocampal RNA (*n* = 3, 3 months) was extracted using miRNeasy mini kit (Qiagen). Tissue was disrupted using a Tissue-Rupter, as per manufacturer’s instructions and resuspended in DNase- and RNase-free water. Total hippocampal RNA sample quality was confirmed by Bioanalyzer (Agilent) and libraries were prepared with the TruSeq RNA v2 LS Kit (Ilumina). RNAseq was then performed employing the HiSeq system (Illumina).

### RNAseq data analysis

Due to the presence of human genes in the Tc1 mouse, a custom reference genome was assembled in order to asses expression levels of Hsa21 genes. A standard mouse genome (NCBI build 37.2) was combined with the Hsa21 sequence (NCBI build 37.2). The RNAseq data was then aligned to this custom genome employing Bowtie (v2.1.0) as part of the Tophat pipeline. Overall count data were then generated, employing the dexseq_count.py script, before combining counts for Hsa21 genes and mouse orthologs. The resulting data, also including non-Hsa21 genes, was then analysed by Deseq to generate adjusted *P* values.

### Quantitative real-time polymerase chain reaction (qPCR)

#### cDNA preparation

For all qPCR experiments, mRNA was sourced from male Tc1 mouse hippocampus (*n* = 4, 6 months). For each sample (labeled WT/Tc1 A–D), 1 μg mRNA was first cleared of genomic DNA (gDNA) via incubation in 2 μl gDNA Wipeout Buffer (2.5–10% trometamol - QIAGEN) and 12 μl RNAse-free ddH_2_0 at 42 °C for 2 min. Following, mRNA was reverse-transcribed into cDNA using a QuantiTect. Reverse Transcription Kit (QIAGEN) according to manufacturer’s instructions. The resulting ~1 μg (50 ng/μl) cDNA product was stored at −20 °C for experimental use. For each sample, an identical reaction was run alongside, substituting the reverse transcriptase with RNAse-free ddH20 for usage as a negative control (−RT) to account for any non-specific amplification at the qPCR stage.

#### Primer and probe design

Exon sequences for AXIN2 and DKK3 were obtained from the e!Ensembl browser (http://www.ensembl.org/index.html) selecting for mouse genome. Primers and probes were designed employing Primer Express® Software (Life Technology) set on TAQman qPCR design. Primers were specifically engineered to bridge exon-exon boundaries. Furthermore, each primer pair and probe was tested for specificity through the NCBI reverse ePCR web service (http://www.ncbi.nlm.nih.gov/projects/e-pcr/forward.cgi). FAM™ and TAMRA™ were employed as fluorophore and quencher, respectively. Upon delivery, primers were solubilised in ddH20 while probes were dissolved in qPCR Probe Dilution Buffer (10 mM Tris-HCl, pH 8, 1 mM EDTA) to a 100 μM stock concentration and stored at −20 °C according to manufacturer’s instructions.

#### qPCR and quantification

For experimental use, primer and probe stocks were diluted to 40 μΜ and 5 μM in ddH20 respectively. Furthermore, each 50 ng/μl cDNA preparation was diluted 1:10 following routine laboratory practice for moderately-to-highly expressed genes. β-actin and glyceraldehyde 3-phosphate dehydrogenase (GAPDH) were employed as control reference genes. Primers and probes for both were labeled with VIC® (Life Technology) and quenched by MGB. All reactions were incubated in TaqMan® Gene Expression Master Mix (AmpliTaq Gold® 55 DNA Polymerase (Ultra Pure), Uracil-DNA glycosylase, dNTPs (with dUTP), ROXTM Passive Reference, and optimised buffer components). Each experiment was performed in a standard 96-well plate format. For all experiments performed in this study, sample WT A was selected as standard. The original 50 ng/μl cDNA preparation was diluted 1:5 and then 1:2 serially up to 1:80. All samples and controls were loaded in technical triplicate. All experiments were performed employing a 7500 Fast Real-Time PCR System (Life Technologies) controlled by homonymous software. The qPCR was ran for a standard duration of approximately 90 min. For all samples, the C_T_ value for each replicate was normalised to the geometric mean of reference gene levels (GAPDH and β-actin). Mean ± SD/SEM were calculated, and all means were then normalised to WT A levels.

#### ULTImate Y2H™ analysis

Yeast two-hybrid screening shown in Fig. 4E was performed by Hybrigenics Services, S.A.S., Paris, France (http://www.hybrigenics-services.com). The coding sequence for full length DYRK1A isoform 1 (NCBI reference (NM_001396.3) was PCR-amplified and cloned into pB27 as a C-terminal fusion to LexA (LexA-DYR1A), and into pB29 as an N-terminal fusion to LexA (DYR1A-LexA). The constructs were checked by sequencing and used as a bait to screen a random-primed Adult Brain cDNA library constructed into pP6. pB27 and pB29 derive from the original pBTM116 vector^98^, and pP6 is based on the pGADGH plasmid^99^. For the N-LexA-DYRK1A-C and the N-DYRK1A-LexA-C bait constructs, 65 million (6.5-fold the complexity of the library) and 115 million (11.5-fold the complexity of the library) clones were screened using a mating approach with YHGX13 (Y187 ade2-101::loxP-kanMX-loxP, matα) and L40ΔGal4 (mat a) yeast strains as previously described^100^. 269 and 253 His+ colonies, respectively, were selected on a medium lacking tryptophan, leucine and histidine and 2 mM 3-AT for N-LexA-DYRK1A-C and 5 mM 3-AT for N-DYRK1A-LexA-C to maintain a strong selectivity and manage the slight autoactivation. The prey fragments of the positive clones were amplified by PCR and sequenced at their 5′ and 3′ junctions. The resulting sequences were used to identify the corresponding interacting proteins in the GenBank database (NCBI) using a fully automated procedure.

#### PBS scoring

A confidence score (PBS, for Predicted Biological Score) was attributed to each interaction. The PBS relies on two different levels of analysis. Firstly, a local score takes into account the redundancy and independency of prey fragments, as well as the distribution of reading frames and stop codons in overlapping fragments. Secondly, a global score takes into account the interactions found in all the screens performed at Hybrigenics using the same library. This global score represents the probability of an interaction being nonspecific. For practical use, the scores were divided into four categories, from A (highest confidence) to D (lowest confidence). A fifth category (E) specifically flags interactions involving highly connected prey domains previously found several times in screens performed on libraries derived from the same organism. Finally, several of these highly connected domains have been confirmed as false-positives of the technique and are now tagged as F. The PBS scores have been shown to positively correlate with the biological significance of interactions^101^.

#### Immunocytochemistry sample preparation

HeLa cells, cells were seeded at ~60% confluency into No. 1.5 imaging chambers (Lab-Tek) coated with 400–600 μg/ml Matrigel (Corning) and transfected immediately using Lipofectamine 3000 (Thermo Fisher Scientific) according to the manufacturer’s specifications. HA-DYRK1A was transfected at 0.25 μg/ml, or else co-transfected with FLAG-DVL1 or FLAG-DKK3 at the same concentration. Wnt3a and LiCl treatment including the appropriate controls were conducted as detailed above 5 hours prior to fixation. After 18 hours cells were fixed with 4% (w/v) paraformaldehyde (PFA) for 20 min at room temperature (RT). Samples were then permeabilised with 0.2% (w/v) saponin (Sigma-Aldrich) for 30 min, and blocked in 5% (v/v) donkey serum (Sigma-Aldrich) doped with 0.05% (w/v) saponin for 1 hour at RT. FLAG and HA primary antibodies (Sigma-Aldrich) were diluted in block to 1:100, added to cells, and incubated overnight at 4 °C. Secondary anti-mouse and anti-rabbit antibodies conjugated to Alexa 488 and Alexa 594 (1:1000; Life Technologies) were made in block and incubated with samples for 30 min at RT. A 1:5000 Hoescht 33258 and 1:1000 Phalloidin 647 (both ThermoFisher Scientific) dilution in PBS was incubated for 30 mins at RT to label the nucleus and F-actin respectively. Cells were thoroughly washed and Imaging was performed in fresh PBS,

HEK293 cells were seeded at ~60% confluency onto glass coverslips previously coated with 0.1 mg/ml poly-D lysine and grown into 6-well plates. Transfection was performed as above but employing 3:1 fuGene HD (Promega) as transfection reagent. Treatment was also performed as above. After 18 hours cells were fixed with 4% (w/v) paraformaldehyde (PFA) for 20 min at RT. Samples were then permeabilised for 30 min in 0.5% (w/v) Triton-X 100, and blocked in 5% (w/v) FBS with 0.05% (w/v) Triton-X 100. The DYRK1A (Abcam) rabbit polyclonal primary antibody was incubated as above at 1:500 dilution. Secondary anti-rabbit Alexa 488 was incubated as above. A 1:1000 DAPI (ThermoFisher Scientific) dilution in block buffer was incubated for 30 mins at RT to label the nucleus.

#### Airyscan and confocal imaging

Airyscan imaging was performed using a Zeiss 880 outfitted with an Airyscan module. Data was collected using a 63 × 1.4 NA objective and immersion oil optimised for 30 °C (Carl Zeiss). Colors were collected sequentially by frame to minimise crosstalk, and Airyscan processing was performed using the Airyscan module in the commercial ZEN software package (Carl Zeiss). Confocal imaging was performed using a Zeiss 710, also using a 63 × 1.4 NA objective and immersion oil.

#### Confocal imaging quantification

Confocal data collected from the Zeiss 710 was used to quantify the nuclear to cytoplasmic ratio of HA-DYRK1A under Wnt stimulation and control conditions. Bitplane (IMARIS) was used to create masks to the nucleus and entire cellular volume. Alexa 488 conjugated HA-DYRK1A fluorescence intensity was integrated across both masks. The nuclear signal was subsequently subtracted from the signal of the entire cellular volume, providing cytoplasmic HA-DYRK1A fluorescence. Data is displayed as a ratio of nuclear to cytoplasmic fluorescence intensity.

#### Western blot and luciferase quantification

Protein bands were quantified from non-processed, raw image files only, directly at the acquisition stage (Syngene GeneGnome system). Automatic background correction was applied to all values before normalising each protein of interest to its relevant β-actin loading control. For phospho/de-phosphoproteins analysed (active β catenin, pS9 GSK3β) values were normalised to total amounts of the relative protein, which were also normalised to β-actin. Values were further normalised to the mean of the control condition (WT for mice, euploid for humans, and empty vector control for cells) for each individual experimental repeat. For all luciferase experiments, TOP/FOPFLASH values quantified alone (Fig. 5A–D) or as a ratio to renilla luciferase activity (Fig. 5E) were normalised to the average control condition in each repeat. Data were then log_2_-transformed prior to analysis. This step was undertaken to more appropriately visualise lucid erase data, due to large fold changes in some cases. In Fig. 5C, the mean normalised signal of treated cells with LiCl +0.1% DMSO was assumed as maximum response, and all other values expressed as percentages of the former. For IVIS experiments (Fig. 5D), preset circular regions of interest (ROIs) were applied to each well, and background was automatically corrected for by subtracting from an average background ROI. These data were then analysed directly without log_2_-transformation, to more appropriately reflect depicted light emission quantified by IVIS. For all sample groups, mean, SD and SEM were calculated.

#### Image processing

In all western blots, no image processing was performed, presenting the blot results in raw format. Raw.sgd files were converted to.tiff and colour-inverted to display background as white/grey. Figures 1, 3, 4 and 5 display horizontally cropped sections of representative blots, and black lines indicate where the image was occasionally cropped vertically (see Fig. S1 for all original, non-cropped blots detailing areas displayed in figures and related loading controls). All co-IP gels in Fig. 4A–D are presented from 1 representative repeat. For IVIS luciferase experiments (Fig. 5D), minimum and maximum display levels were automatically set by the system according to measured signal, independent of quantification.

### Ingenuity pathway analysis

The Qiagen Ingenuity® Pathway Analysis (IPA®) tool was employed to probe mouse and human RNAseq datasets for alterations in primary Wnt components and upstream regulators. For Fig. 3B–D, Tc1 RNAseq analysis was performed on a dataset of significantly altered genes at adjusted *P* < 0.05. In all cases, IPA analysis parameters were left at default, searching only for direct relationships and for human, mouse and rat orthologs. For each dataset, pathway, upstream regulator and diseases/functions analysis were performed. Curated gene lists were produced by manually selecting all affected genes in the dataset indicated as primary Wnt components, or under regulation of β-catenin/Wnt3a. Curated lists of upstream regulators were generated by searching for Wnt-related proteins contained in the analysis output. Z-scores and molecular activity predictions (MAPs) were generated automatically by IPA.

### STRING and cytoscape network analysis

The protein interaction database and analysis tool STRING v10.0^58^ was employed to build the networks in Fig. 4E,F. To achieve this goal, DYRK1A was placed as the hub of a basic network (Fig. 4E, red circles) comprising: (1) The Hybrigenics-reported DKK3 interaction; (2) Previously published interactions (solid black lines) between DYRK1A, p-120 catenin (CTNND1)^31^, and GSK3β^35^; (C) Experimentally detected DKK3 and DVL1 interactions (dotted black lines). Components of the basic network in Fig. 4E were used as starting nodes, selecting human as default organism. This initial network, or first shell, was enriched with the 10 best-scoring interactors. A further shell was then added, comprising the 10 best-scoring secondary interactors of the network. The network diameter, or distance between each node, was limited to 3, meaning that each node is no more than one degree of interaction away from any other. This was done to better reveal a highly interconnected network. The resultant network (Fig. 4F) has 140 edges and 25 nodes. The network was then imported into the Cytoscape network analysis tool and its visual properties were modified to distinguish amongst node types and visually aid description of the results. The network was displayed employing a spring-embedded layout, manually adjusted to reflect the known structure of canonical Wnt signalling.

### Ethics approval and consent to participate

The use of animals was conducted in line with the ethical principles of Replacement, Reduction and Refinement (3Rs). All animal studies were approved by the University College London Animal Welfare and Ethical Review Body (AWERB) and licensed by the home office.

## Supplementary information

Supplementary Figures

Supplementary Table 1

Supplementary Table 2

Supplementary Table 3

Supplementary Table 4

Supplementary Table 5

Supplementary Table 6

## Data Availability

All raw data are available on request.

## References

[1] Sherman SL, Allen EG, Bean LH, Freeman SB (2007). Epidemiology of Down syndrome. Ment Retard Dev Disabil Res Rev.

[2] Hernandez D, Fisher EM (1996). Down syndrome genetics: unravelling a multifactorial disorder. Hum Mol Genet.

[3] Bittles AH, Glasson EJ (2004). Clinical, social, and ethical implications of changing life expectancy in Down syndrome. Dev Med Child Neurol.

[4] Natoli JL, Ackerman DL, McDermott S, Edwards JG (2012). Prenatal diagnosis of Down syndrome: a systematic review of termination rates (1995–2011). Prenat Diagn.

[5] Ivan DL, Cromwell P (2014). Clinical practice guidelines for management of children with Down syndrome: Part I. J Pediatr Health Care.

[6] Letourneau A (2014). Domains of genome-wide gene expression dysregulation in Down’s syndrome. Nature.

[7] Lejeune JTRGM, Turpin R, Gautier M (1959). Le mongolisme, premier exemple d’aberration autosomique humaine. Ann Genet.

[8] Kahlem P (2006). Gene-dosage effect on chromosome 21 transcriptome in trisomy 21: implication in Down syndrome cognitive disorders. Behav Genet.

[9] Nusse R, Varmus H (2012). Three decades of Wnts: a personal perspective on how a scientific field developed. The EMBO journal.

[10] Fuerer C, Nusse R, Ten Berge D (2008). Wnt signalling in development and disease. Max Delbruck Center for Molecular Medicine meeting on Wnt signaling in Development and Disease. EMBO Rep.

[11] Inestrosa NC, Varela-Nallar L (2015). Wnt signalling in neuronal differentiation and development. Cell Tissue Res.

[12] Inestrosa NC, Arenas E (2010). Emerging roles of Wnts in the adult nervous system. Nature Rev Neurosci.

[13] Cordero JB, Sansom OJ (2012). Wnt signalling and its role in stem cell-driven intestinal regeneration and hyperplasia. Acta Physiol (Oxf).

[14] Anastas JN, Moon RT (2013). WNT signalling pathways as therapeutic targets in cancer. Nat Rev Cancer.

[15] Wisniewski KE, Wisniewski HM, Wen GY (1985). Occurrence of neuropathological changes and dementia of Alzheimer’s disease in Down’s syndrome. Ann Neurol.

[16] Leverenz JB, Raskind MA (1998). Early amyloid deposition in the medial temporal lobe of young Down syndrome patients: a regional quantitative analysis. Exp neurol.

[17] Wiseman FK (2015). A genetic cause of Alzheimer disease: mechanistic insights from Down syndrome. Nat Rev Neurosci.

[18] Toledo EM, Inestrosa NC (2010). Wnt signaling activation reduces neuropathological markers in a mouse model of Alzheimer’s disease. Mol Psychiatry.

[19] Inestrosa NC, Montecinos-Oliva C, Fuenzalida M (2012). Wnt signaling: role in Alzheimer disease and schizophrenia. J Neuroimmune Pharmacol.

[20] Purro SA, Dickins EM, Salinas PC (2012). The secreted Wnt antagonist Dickkopf-1 is required for amyloid β-mediated synaptic loss. J Neurosci.

[21] Skaper SD (2014). Wnt-signalling: A new direction for alzheimer disease?. CNS Neurol Disord Drug Targets.

[22] Tiwari SK (2015). Ethosuximide Induces Hippocampal Neurogenesis and Reverses Cognitive Deficits in an Amyloid-beta Toxin-induced Alzheimer Rat Model via the Phosphatidylinositol 3-Kinase (PI3K)/Akt/Wnt/beta-Catenin Pathway. J Biol Chem.

[23] Kim DY, Jung SY, Kim K, Kim CJ (2016). Treadmill exercise ameliorates Alzheimer disease-associated memory loss through the Wnt signaling pathway in the streptozotocin-induced diabetic rats. J Exerc Rehabil.

[24] Vallee A, Lecarpentier Y (2016). Alzheimer Disease: Crosstalk between the Canonical Wnt/Beta-Catenin Pathway and PPARs Alpha and Gamma. Front Neurosci.

[25] Arron JR (2006). NFAT dysregulation by increased dosage of DSCR1 and DYRK1A on chromosome 21. Nature.

[26] Fernandez-Martinez J (2009). Attenuation of Notch signalling by the Down-syndrome-associated kinase DYRK1A. J Cell Sci.

[27] Park J, Song WJ, Chung KC (2009). Function and regulation of Dyrk1A: towards understanding Down syndrome. Cell Mol Life Sci.

[28] Scales TM, Lin S, Kraus M, Goold RG, Gordon-Weeks PR (2009). Nonprimed and DYRK1A-primed GSK3 beta-phosphorylation sites on MAP1B regulate microtubule dynamics in growing axons. J Cell Sci.

[29] Becker W (2011). Recent insights into the function of DYRK1A. FEBS J.

[30] Hammerle B (2011). Transient expression of Mnb/Dyrk1a couples cell cycle exit and differentiation of neuronal precursors by inducing p27(KIP1) expression and suppressing NOTCH signaling. Development.

[31] Hong JY (2012). Down’s-syndrome-related kinase Dyrk1A modulates the p120-catenin/Kaiso trajectory of the Wnt signaling pathway. J cell sci.

[32] Tlili A (2013). Hepatocyte-specific Dyrk1a gene transfer rescues plasma apolipoprotein A-I levels and aortic Akt/GSK3 pathways in hyperhomocysteinemic mice. Biochim Biophys Acta.

[33] Booiman T, Loukachov VV, van Dort KA, van ‘t Wout AB, Kootstra NA (2015). DYRK1A Controls HIV-1 Replication at a Transcriptional Level in an NFAT Dependent Manner. PLoS One.

[34] Najas S (2015). DYRK1A-mediated Cyclin D1 Degradation in Neural Stem Cells Contributes to the Neurogenic Cortical Defects in Down Syndrome. EBioMedicine.

[35] Song W-J (2015). Phosphorylation and inactivation of glycogen synthase kinase 3β (GSK3β) by dual-specificity tyrosine phosphorylation-regulated kinase 1A (Dyrk1A). J Biol Chem.

[36] Oi A (2017). Subcellular distribution of cyclin-dependent kinase-like 5 (CDKL5) is regulated through phosphorylation by dual specificity tyrosine-phosphorylation-regulated kinase 1A (DYRK1A). Biochem Biophys Res Commun.

[37] Kimura R (2007). The DYRK1A gene, encoded in chromosome 21 Down syndrome critical region, bridges between β-amyloid production and tau phosphorylation in Alzheimer disease. Hum mol gen.

[38] Park J, Yang EJ, Yoon JH, Chung KC (2007). Dyrk1A overexpression in immortalized hippocampal cells produces the neuropathological features of Down syndrome. Mol Cell Neurosci.

[39] Ryoo SR (2007). DYRK1A-mediated hyperphosphorylation of Tau. A functional link between Down syndrome and Alzheimer disease. J Biol Chem.

[40] Ryoo SR (2008). Dual-specificity tyrosine(Y)-phosphorylation regulated kinase 1A-mediated phosphorylation of amyloid precursor protein: evidence for a functional link between Down syndrome and Alzheimer’s disease. J Neurochem.

[41] Shi J (2008). Increased dosage of Dyrk1A alters alternative splicing factor (ASF)-regulated alternative splicing of tau in Down syndrome. J Biol Chem.

[42] Ryu YS (2010). Dyrk1A-mediated phosphorylation of Presenilin 1: a functional link between Down syndrome and Alzheimer’s disease. J Neurochem.

[43] Wegiel J (2011). Link Between DYRK1A Overexpression and Several-Fold Enhancement of Neurofibrillary Degeneration With 3-Repeat Tau Protein in Down Syndrome. J Neuropathol Exp Neurol.

[44] Janel N, Sarazin M, Corlier F, Corne H, de Souza L C, Hamelin L, Aka A, Lagarde J, Blehaut H, Hindié V, Rain J-C, Arbones M L, Dubois B, Potier M C, Bottlaender M, Delabar J M (2014). Plasma DYRK1A as a novel risk factor for Alzheimer’s disease. Translational Psychiatry.

[45] Coutadeur S (2015). A novel DYRK1A (dual specificity tyrosine phosphorylation-regulated kinase 1A) inhibitor for the treatment of Alzheimer’s disease: effect on Tau and amyloid pathologies *in vitro*. J Neurochem.

[46] Berwick DC (2017). Pathogenic LRRK2 variants are gain-of-function mutations that enhance LRRK2-mediated repression of beta-catenin signaling. Mol Neurodegener.

[47] O’Doherty A (2005). An aneuploid mouse strain carrying human chromosome 21 with Down syndrome phenotypes. Science.

[48] Gribble SM (2013). Massively parallel sequencing reveals the complex structure of an irradiated human chromosome on a mouse background in the Tc1 model of Down syndrome. PLoS One.

[49] Lana-Elola, E. *et al*. Genetic dissection of Down syndrome-associated congenital heart defects using a new mouse mapping panel. *Elife* **5**, 10.7554/eLife.11614 (2016). 10.7554/eLife.11614 PMC4764572 26765563

[50] Choong, X. Y., Tosh, J. L., Pulford, L. J. & Fisher, E. Dissecting Alzheimer disease in Down syndrome using mouse models. *Front Behav Neurosci* **9** (2015). 10.3389/fnbeh.2015.00268 PMC4602094 26528151

[51] Kramer A, Green J, Pollard J, Tugendreich S (2014). Causal analysis approaches in Ingenuity Pathway Analysis. Bioinformatics.

[52] Sheppard Olivia, Plattner Florian, Rubin Anna, Slender Amy, Linehan Jacqueline M., Brandner Sebastian, Tybulewicz Victor L.J., Fisher Elizabeth M.C., Wiseman Frances K. (2012). Altered regulation of tau phosphorylation in a mouse model of down syndrome aging. Neurobiology of Aging.

[53] Ahmed MM (2013). Protein profiles in Tc1 mice implicate novel pathway perturbations in the Down syndrome brain. Hum Mol Genet.

[54] Yu, T. *et al*. A mouse model of Down syndrome trisomic for all human chromosome 21 syntenic regions. *Hum mol gen* **179** (2010). 10.1093/hmg/ddq179 PMC2893810 20442137

[55] Malliri A (2006). The rac activator Tiam1 is a Wnt-responsive gene that modifies intestinal tumor development. J Biol Chem.

[56] Koh SH (2007). Inhibition of glycogen synthase kinase-3 suppresses the onset of symptoms and disease progression of G93A-SOD1 mouse model of ALS. Exp Neurol.

[57] Zhou F (2012). The APP intracellular domain (AICD) inhibits Wnt signalling and promotes neurite outgrowth. Biochim Biophys Acta.

[58] Szklarczyk D (2015). String v10: protein-protein interaction networks, integrated over the tree of life. Nucleic Acids Res.

[59] Álvarez M, Estivill X, de la Luna S (2003). DYRK1A accumulates in splicing speckles through a novel targeting signal and induces speckle disassembly. J cell sci.

[60] Torre R (2014). Epigallocatechin‐3‐gallate, a DYRK1A inhibitor, rescues cognitive deficits in Down syndrome mouse models and in humans. Mol Nutr Food Res.

[61] Ogawa Y (2010). Development of a novel selective inhibitor of the Down syndrome-related kinase Dyrk1A. Nat Commun.

[62] Adayev T, Wegiel J, Hwang Y-W (2011). Harmine is an ATP-competitive inhibitor for dual-specificity tyrosine phosphorylation-regulated kinase 1A (Dyrk1A). Arch Biochem Biophys.

[63] Contestabile A (2013). Lithium rescues synaptic plasticity and memory in Down syndrome mice. J Clin Invest.

[64] Hammerle B (2003). Expression patterns and subcellular localization of the Down syndrome candidate protein MNB/DYRK1A suggest a role in late neuronal differentiation. Eur J Neurosci.

[65] Hammerle B, Elizalde C, Tejedor FJ (2008). The spatio-temporal and subcellular expression of the candidate Down syndrome gene Mnb/Dyrk1A in the developing mouse brain suggests distinct sequential roles in neuronal development. Eur J Neurosci.

[66] Schwarz-Romond T, Merrifield C, Nichols BJ, Bienz M (2005). The Wnt signalling effector Dishevelled forms dynamic protein assemblies rather than stable associations with cytoplasmic vesicles. J cell sci.

[67] Sear RP (2007). Dishevelled: a protein that functions in living cells by phase separating. Soft Matter.

[68] Rachidi M, Lopes C (2008). Mental retardation and associated neurological dysfunctions in Down syndrome: a consequence of dysregulation in critical chromosome 21 genes and associated molecular pathways. Eur J Paediatr Neurol.

[69] Antonarakis SE (2017). Down syndrome and the complexity of genome dosage imbalance. Nat Rev Genet.

[70] Olmos-Serrano JL (2016). Down Syndrome Developmental Brain Transcriptome Reveals Defective Oligodendrocyte Differentiation and Myelination. Neuron.

[71] Sullivan, K. D. *et al*. Trisomy 21 consistently activates the interferon response. *Elife* **5**, 10.7554/eLife.16220 (2016). 10.7554/eLife.16220 PMC5012864 27472900

[72] Diep DB, Hoen N, Backman M, Machon O, Krauss S (2004). Characterisation of the Wnt antagonists and their response to conditionally activated Wnt signalling in the developing mouse forebrain. Dev Brain Res.

[73] Zenzmaier C, Marksteiner J, Kiefer A, Berger P, Humpel C (2009). Dkk3 is elevated in CSF and plasma of Alzheimer’s disease patients. J Neurochem.

[74] Reya T, Clevers H (2005). Wnt signalling in stem cells and cancer. Nature.

[75] McNeill H, Woodgett JR (2010). When pathways collide: collaboration and connivance among signalling proteins in development. Nat Rev Mol Cell Biol.

[76] Woods YL (2001). The kinase DYRK phosphorylates protein-synthesis initiation factor eIF2Bepsilon at Ser539 and the microtubule-associated protein tau at Thr212: potential role for DYRK as a glycogen synthase kinase 3-priming kinase. Biochem. J.

[77] Arron JR (2006). NFAT dysregulation by increased dosage of DSCR1 and DYRK1A on chromosome 21. Nature.

[78] Kurabayashi N, Hirota T, Sakai M, Sanada K, Fukada Y (2010). DYRK1A and glycogen synthase kinase 3β, a dual-kinase mechanism directing proteasomal degradation of CRY2 for circadian timekeeping. Mol Cell Biol.

[79] Shen W (2015). Inhibition of DYRK1A and GSK3B induces human beta-cell proliferation. Nat Commun.

[80] Leonard JL (2017). The Dkk3 gene encodes a vital intracellular regulator of cell proliferation. PLoS One.

[81] Caricasole A (2004). Induction of Dickkopf-1, a negative modulator of the Wnt pathway, is associated with neuronal degeneration in Alzheimer’s brain. J Neurosci.

[82] Purro, S. A., Galli, S. & Salinas, P. C. Dysfunction of Wnt signaling and synaptic disassembly in neurodegenerative diseases. *Journal of molecular cell biology*, *mjt0* **49** (2014). 10.1093/jmcb/mjt049 PMC4344549 24449494

[83] Lie DC (2005). Wnt signalling regulates adult hippocampal neurogenesis. Nature.

[84] Seib DR (2013). Loss of Dickkopf-1 restores neurogenesis in old age and counteracts cognitive decline. Cell Stem Cell.

[85] Chen J, Park CS, Tang SJ (2006). Activity-dependent synaptic Wnt release regulates hippocampal long term potentiation. J Biol Chem.

[86] Stewart DJ (2014). Wnt signaling pathway in non-small cell lung cancer. J Natl Cancer Inst.

[87] Sullivan SG, Hussain R, Glasson EJ, Bittles AH (2007). The profile and incidence of cancer in Down syndrome. J Intellect Disabil Res.

[88] Malinge S (2012). Increased dosage of the chromosome 21 ortholog Dyrk1a promotes megakaryoblastic leukemia in a murine model of Down syndrome. The Journal of clinical investigation.

[89] Xue G, Romano E, Massi D, Mandala M (2016). Wnt/beta-catenin signaling in melanoma: Preclinical rationale and novel therapeutic insights. Cancer Treat Rev.

[90] Yang K (2016). The evolving roles of canonical WNT signaling in stem cells and tumorigenesis: implications in targeted cancer therapies. Lab Invest.

[91] Harvey K, Marchetti B (2014). Regulating Wnt signaling: a strategy to prevent neurodegeneration and induce regeneration. J Mol Cell Biol.

[92] Wan W, Xia S, Kalionis B, Liu L, Li Y (2014). The role of Wnt signaling in the development of Alzheimer’s disease: a potential therapeutic target?. Biomed Res Int.

[93] Becker W, Soppa U, Tejedor FJ (2014). DYRK1A: a potential drug target for multiple Down syndrome neuropathologies. CNS Neurol Disord Drug Targets.

[94] Stagni F (2017). Epigallocatechin gallate: A useful therapy for cognitive disability in Down syndrome?. Neurogenesis (Austin).

[95] Sancho RM, Law BM, Harvey K (2009). Mutations in the LRRK2 Roc-COR tandem domain link Parkinson’s disease to Wnt signalling pathways. Hum Mol Genet.

[96] Veeman MT, Slusarski DC, Kaykas A, Louie SH, Moon RT (2003). Zebrafish prickle, a modulator of noncanonical Wnt/Fz signaling, regulates gastrulation movements. Curr Biol.

[97] Nixon-Abell, J. *et al*. Protective LRRK2 R1398H variant enhances GTPase and Wnt signaling activity. *Front Mol Neurosci* **9** (2016). 10.3389/fnmol.2016.00018 PMC4781896 27013965

[98] Vojtek AB, Hollenberg SM (1995). Ras-Raf interaction: two-hybrid analysis. Methods Enzymol.

[99] Bartel P, Chien CT, Sternglanz R, Fields S (1993). Elimination of false positives that arise in using the two-hybrid system. Biotechniques.

[100] Fromont-Racine M, Rain JC, Legrain P (1997). Toward a functional analysis of the yeast genome through exhaustive two-hybrid screens. Nat Genet.

[101] Rain JC (2001). The protein-protein interaction map of Helicobacter pylori. Nature.

