# Supplementary material for: Downregulated Wnt/β-catenin signalling in the Down syndrome hippocampus
Source: Sci Rep. 2019 May 13;9:7322. doi: 10.1038/s41598-019-43820-4 (PMC6513850; doi:10.1038/s41598-019-43820-4)
Supplement: Supplementary file 1 — Supplementary Figures [file 41598_2019_43820_MOESM1_ESM.pdf]

Supplementary Data

Downregulated Wnt/β-catenin signalling in the Down syndrome hippocampus

Simone Granno*[1,2], Jonathon Nixon-Abell*[1,3], Daniel C. Berwick[1,4], Justin Tosh[2], George Heaton[1], Sultan Almudimeegh[1], Zenisha Nagda[1], Jean-Christophe Rain[5], Manuela Zanda[6], Vincent Plagnol[6], Victor L.J. Tybulewicz[7,8,9], Karen Cleverley[2], Frances K. Wiseman[2, 9], Elizabeth M.C. Fisher[2,9] & Kirsten Harvey▾[1]

Affiliations

[1]Department of Pharmacology, UCL School of Pharmacy, University College London, 29-39 Brunswick Square, London WC1N 1AX, UK.

[2]Department of Neuromuscular Diseases, UCL Institute of Neurology, Queen Square, London WC1N 3BG, UK

[3]Cell Biology Section, Neurogenetics Branch, National Institute of Neurological Disorders and Stroke (NINDS), Bethesda, MD, USA.

[4]School of Health, Life and Chemical Sciences, The Open University, Walton Hall, Milton Keynes MK6 7AA, UK.

[5]Hybrigenics Services - Fondation Jérôme Lejeune, 3-5 Impasse Reille 75014 Paris, France

[6]UCL Genetics Institute, Darwin Building, Gower Street, London WC1E 6BT, UK

[7]The Francis Crick Institute, 1 Midland Rd, Kings Cross, London NW1 1AT, UK

[8]Department of Medicine, Imperial College, London W12 0NN, UK

[9]London Down Syndrome Consortium (LonDownS)

*These authors contributed equally to the study

▾Correspondence: kirsten.harvey@ucl.ac.uk,

Department of Pharmacology, UCL School of Pharmacy, University College London, 29-39 Brunswick Square, London WC1N 1AX, UK

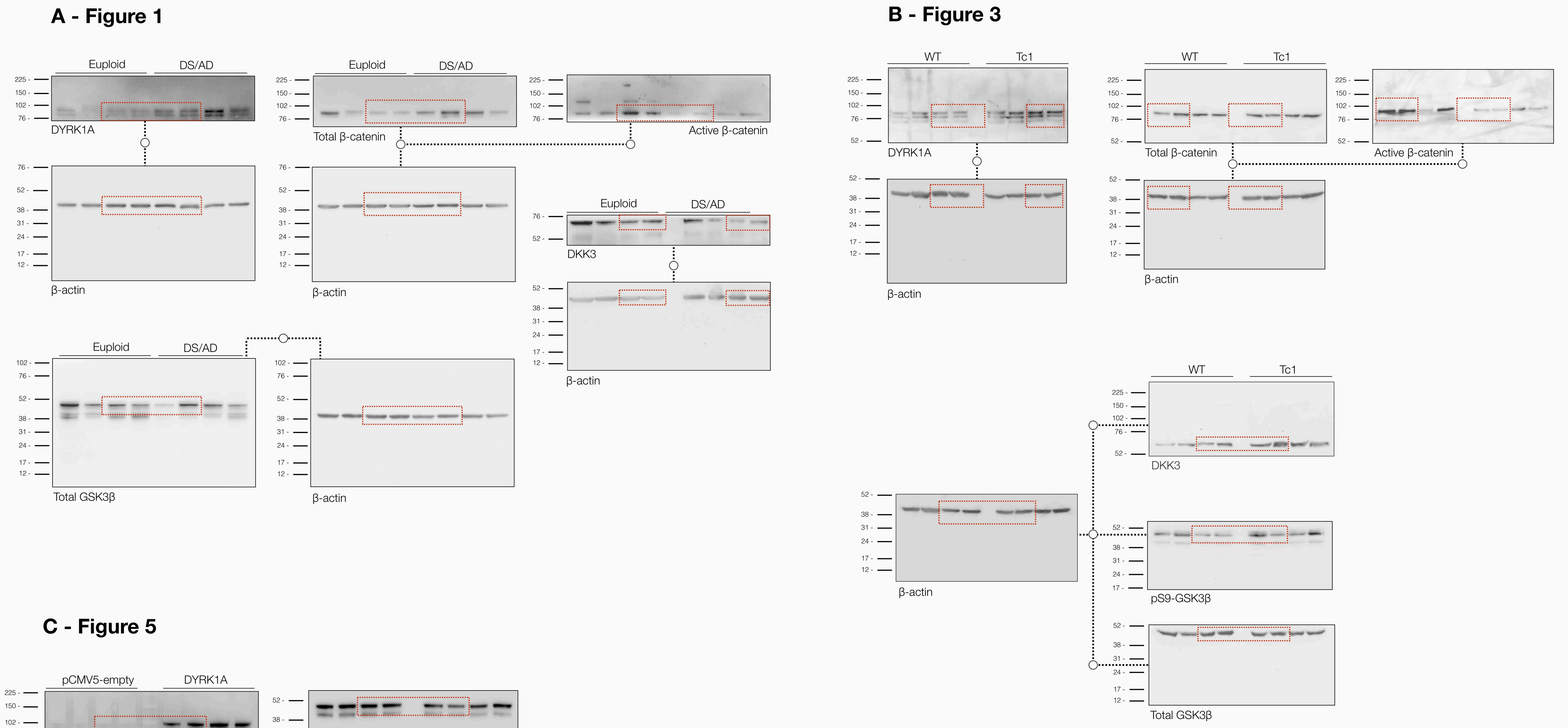

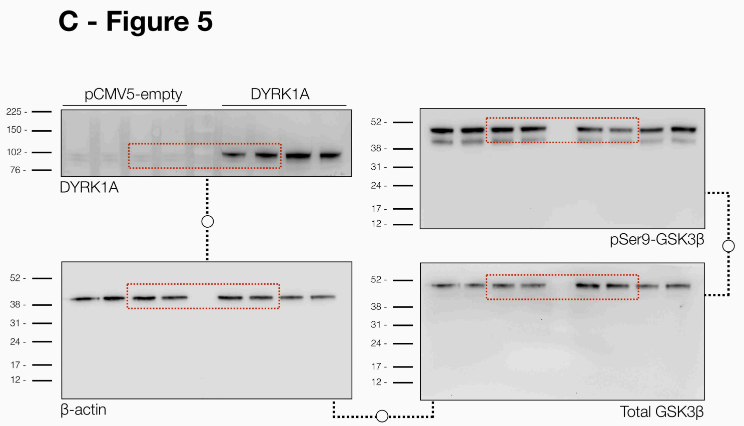

Fig. S1 - Whole, uncropped representative Western blots for all figures displaying immunoblotting data. Highlighted areas (red dashed lines) represent cropped portion used for main figures. Connection line demonstrate which β-actin control is associated with each protein of interest in the main figures. Ladder on left hand side of each gel represent molecular weight marker in kDa.

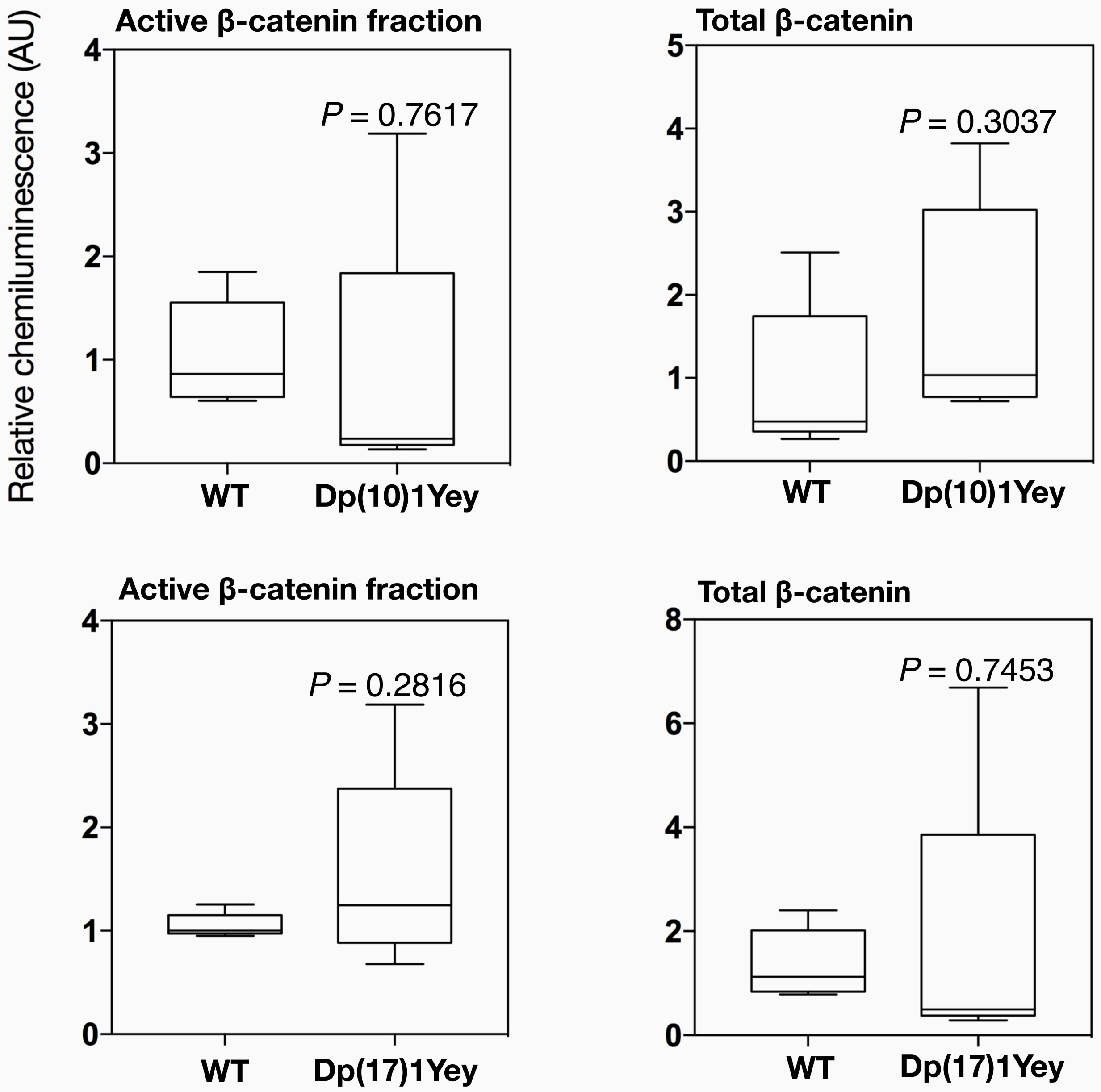

Fig. S2 - Total and active β-catenin levels in the Dp(10)1Yey (top) and Dp(17)1Yey (bottom) mouse hippocampus, demonstrating no significant differences in Wnt signalling activity in these models.

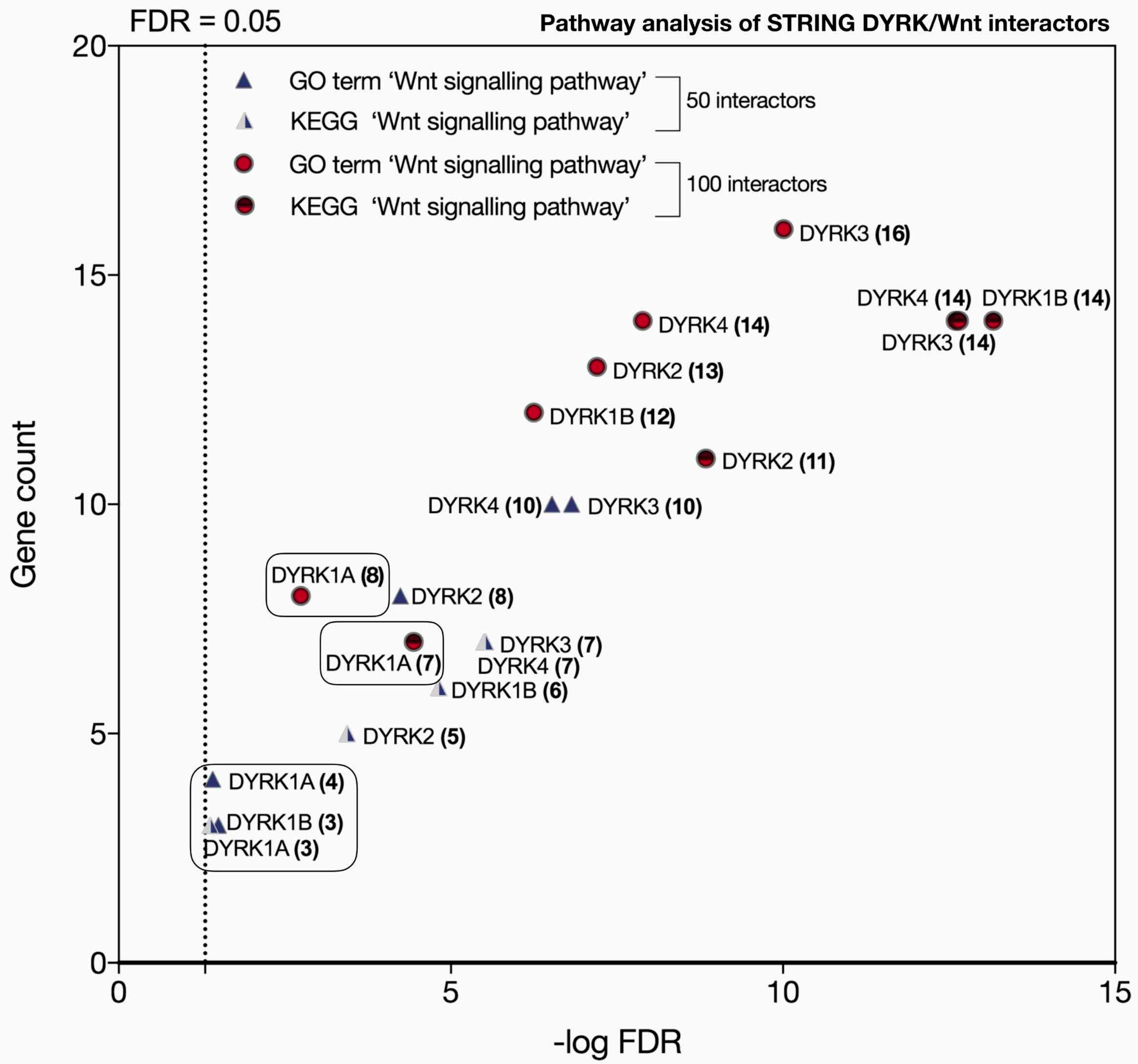

Fig. S3 - Gene ontology (GO) and KEGG pathway associations derived from a literature-mined interaction network generated by STRING for all DYRK family members, searching for DYRKs alone and adding 50 direct interactors (triangles) or a further 50 secondary interactors (circles), demonstrating a significant association with the Wnt signalling pathway. Gene counts for each DYRK are plotted against significance ($-\log_{10}P$).

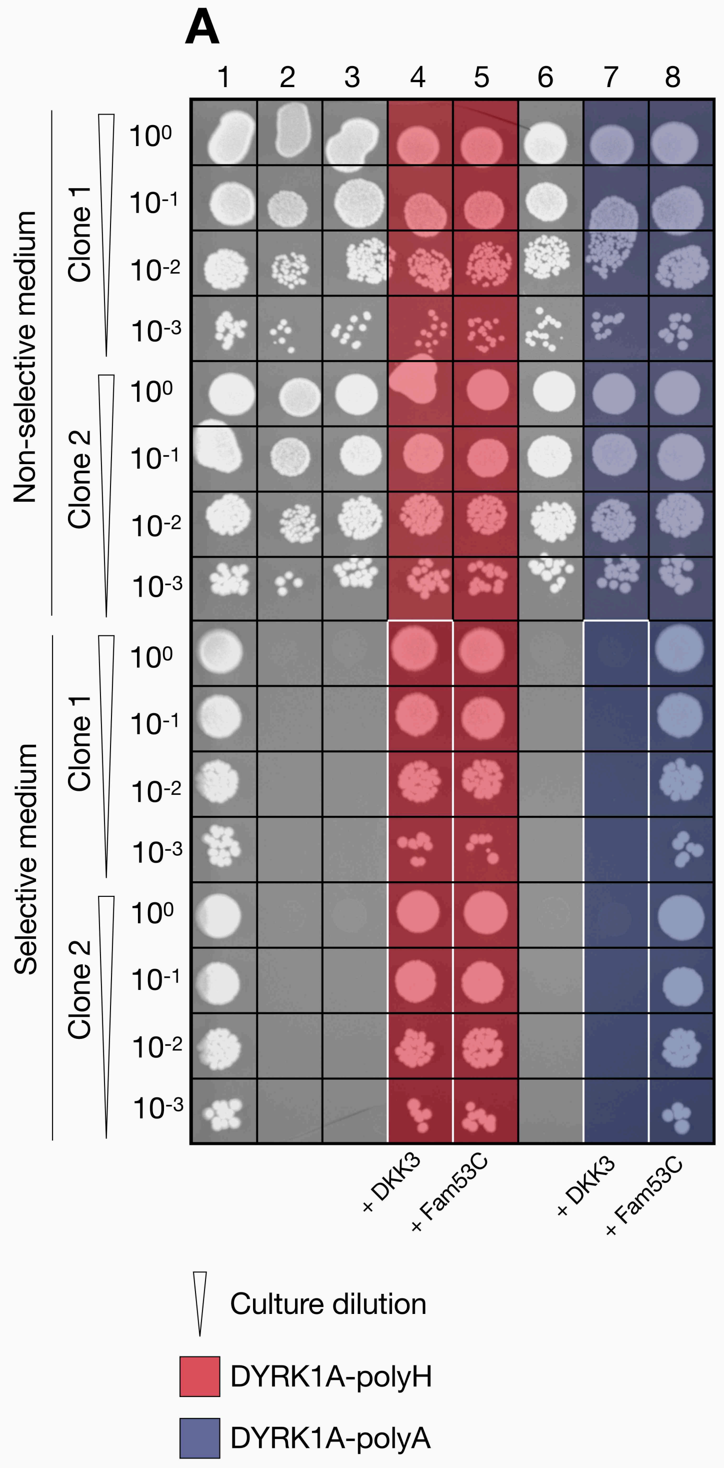

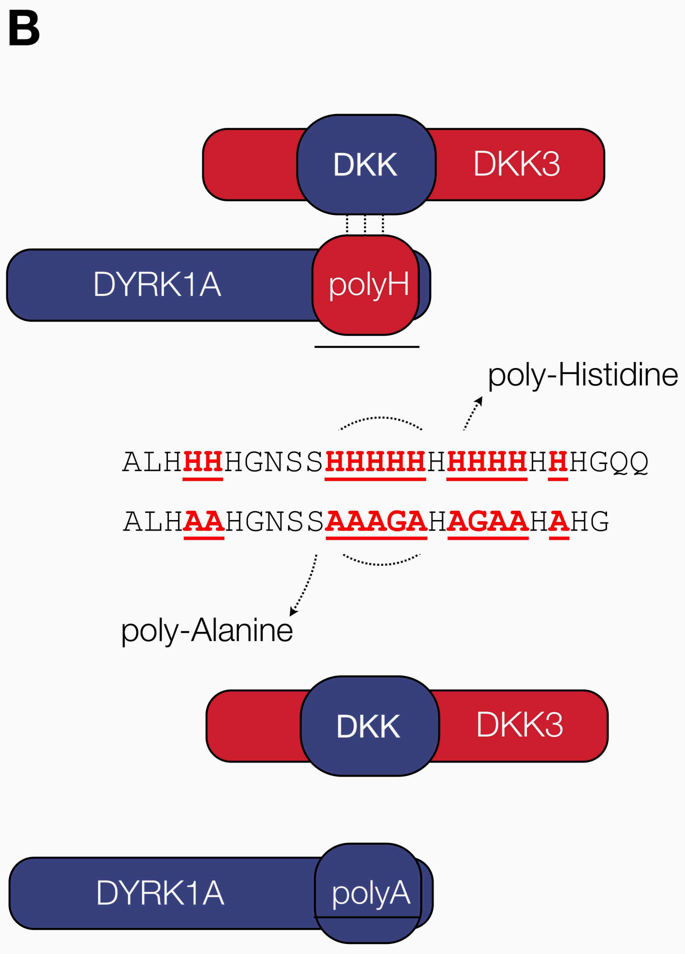

Fig. S4 - The DKK3-DYRK1A interaction is dependent on the poly histidine tail of DYRK1A. **(A)** Confirmatory Y2H assay demonstranting a strong DKK3-DYRK1A interaction, which is abolished by mutation of the poly-histidine stretch of DYRK1A (lanes 7-8): **1** Positive Control (Smad/Smurf), **2** Negative Control (lexA-ø/AD-ø), **3** Negative Control (LexA-Dyrk1A /AD-ø), **4** Interaction DYRK1A/DKK3 aa131-aa245, **5** Interaction DYRK1A/Fam53C aa339-452, **6** Negative Control (LexA-DYRK1A-poly-Ala/AD-ø), **7** Interaction LexA-DYRK1A-poly-Ala/ DKK3 aa131-aa245 **8** Interaction LexA-DYRK1A-poly-His/Fam53C. **(B)** Graphical summary depicting mutation sequence in **A** (lanes 7-8).

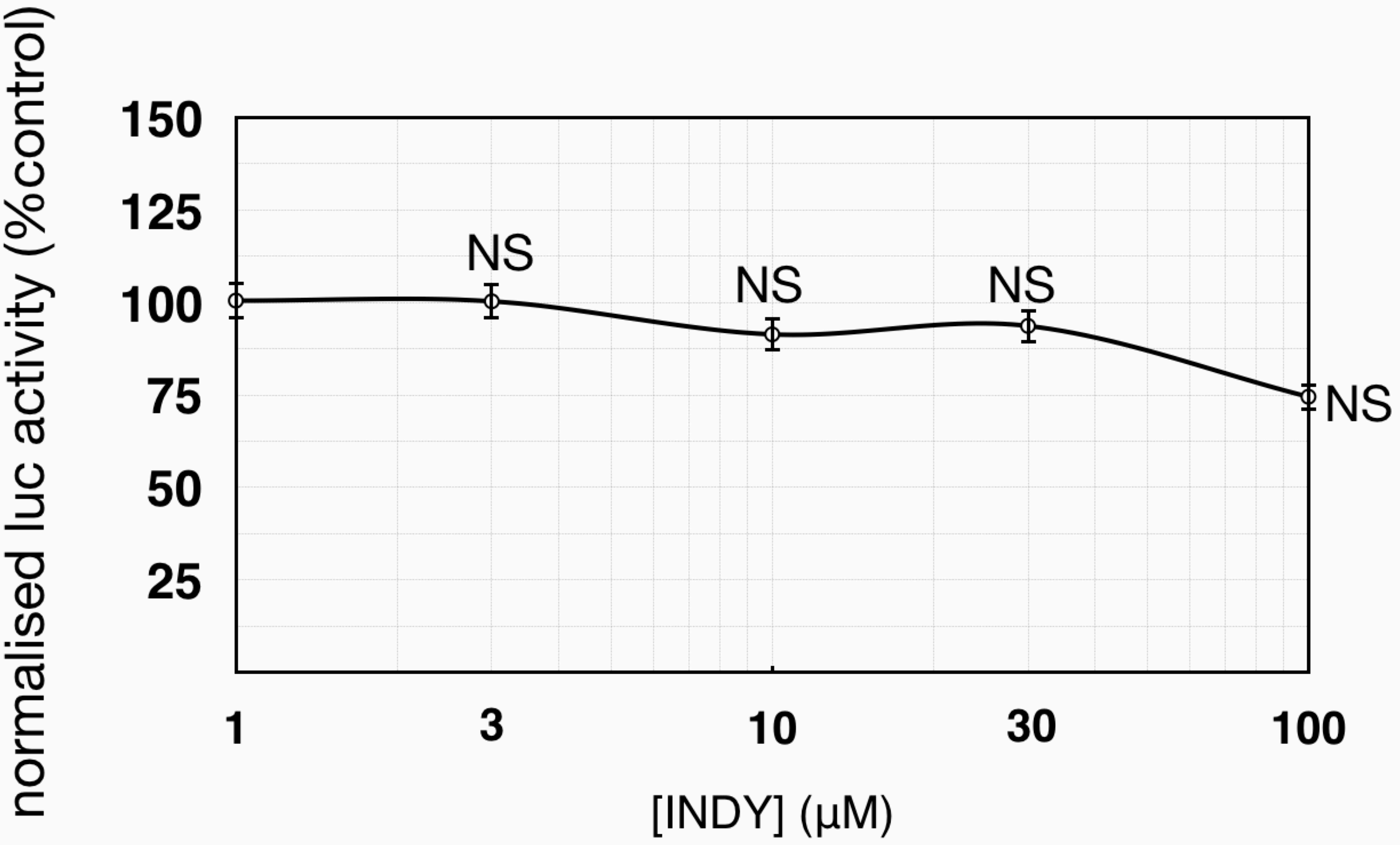

Fig. S5 - No effect of incremental doses of INDY on basal Wnt signalling activity. SH-SY5Y cells stably expressing the TCF-LEF luciferase reporter were treated with 40 mM NaCl, and doses of 1-100 μM INDY were administered for 5 hours. Luciferase activity is plotted as percentage of control treatment.

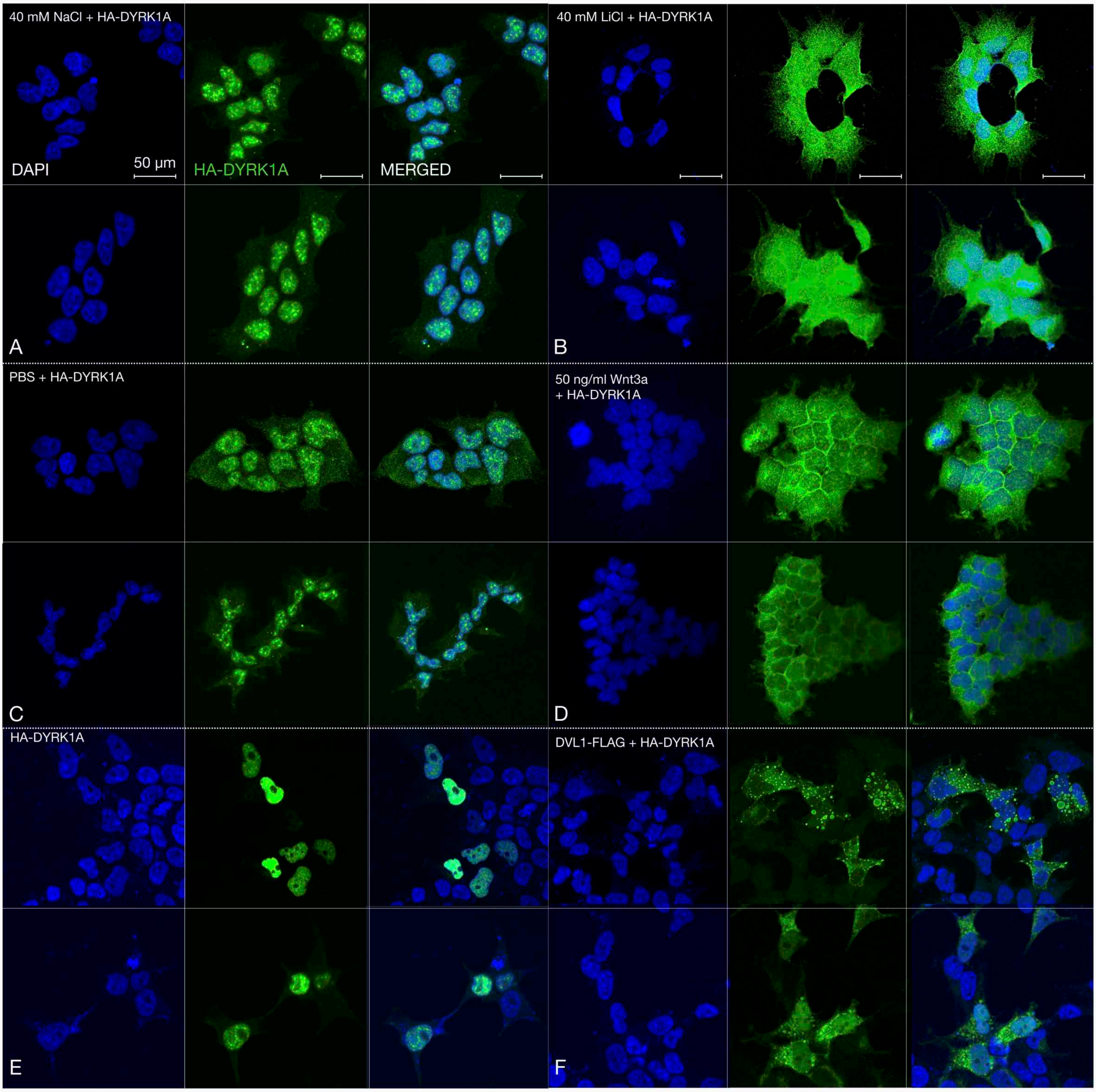

Fig. S6 - Confocal imaging in HEK293 cells, reproducing conditions in fig. 6 for quantification purposes. **(A-B)** Same as fig. 6A, reflecting quantification in fig.6B. **(C-D)** Same as fig.6C, reflecting quantification in fig.6D. **(E-F)** Same as fig.6E, reflecting quantification shown in fig.6F.
